# Disruption of chitin synthases impairs tick feeding and reproduction, validating a broad-spectrum acaricide target

**DOI:** 10.3389/fcimb.2026.1822003

**Published:** 2026-05-15

**Authors:** Ondrej Hajdusek, Veronika Urbanova, Martin Moos, Marie Vancová, Jan Perner

**Affiliations:** 1 Institute of Parasitology, Biology Centre, Czech Academy of Sciences, Ceske Budejovice, Czechia; 2 Institute of Entomology, Biology Centre, Czech Academy of Sciences, Ceske Budejovice, Czechia; 3 Faculty of Agriculture and Technology, University of South Bohemia, Ceske Budejovice, Czechia

**Keywords:** acaricide, Borrelia, chitin, chitin synthase, etoxazole, resistance, RNAi, tick

## Abstract

Chitin, a major structural component of arthropods, is synthesized by chitin synthases (CHSs), which polymerize N-acetylglucosamine and extrude chitin into the extracellular matrix. CHSs are essential for cuticle formation and peritrophic matrix (PM) biogenesis and represent validated targets of several acaricides, yet their roles in ticks remain poorly defined. Here, we functionally characterize CHSs in the tick *Ixodes ricinus*, a major European vector of Lyme borreliosis. We identify three *CHS* genes with distinct expression patterns. RNA interference-mediated silencing of individual *CHS* genes reduced chitin deposition in the cuticle and PM, impaired feeding, survival, molting, and oviposition, and caused high post-feeding mortality. Artificial feeding with etoxazole, a non-catalytic CHS inhibitor, phenocopied these defects at nanomolar concentrations. While *CHS* silencing did not affect *Borrelia afzelii* transmission, it increased pathogen acquisition by feeding nymphs. Together, our findings establish CHSs as essential regulators of tick physiology and highlight their potential as selective molecular targets for tick control strategies.

## 1 Introduction

Ticks, close relatives of mites and spiders within the chelicerate arthropods, are notorious ectoparasites with substantial impacts on human and animal health. They are considered among the most economically significant ectoparasites of livestock and cause considerable financial losses worldwide, amounting to billions of US dollars annually (Jongejan and Uilenberg, 2004; Sonenshine and Roe, 2014). Tick infestations lead to reduced milk and meat production, anemia, secondary infections and, in severe cases, death of infested animals. In addition to the harmful effects of tick feeding, ticks also act as vectors for numerous human and animal pathogens, including viruses (e.g., tick-borne encephalitis virus), bacteria (e.g., *Borrelia*, *Anaplasma*, *Rickettsia*) and protozoa (e.g., *Theileria*, *Babesia*) (de la Fuente et al., 2008; Hajdušek et al., 2013).

Current tick-control strategies rely heavily on chemical acaricides, including synthetic pyrethroids (e.g., permethrin, which disrupts voltage-gated sodium channels in the tick nervous system), macrocyclic lactones (e.g., ivermectin, which blocks glutamate-gated chloride channels), formamidines (e.g., amitraz, an octopamine receptor agonist), and benzoylphenylurea compounds (e.g., fluazuron, an insect growth regulator (IGR)) (Obaid et al., 2022). Depending on their mode of action, these compounds are administered to animals either topically, where they act as contact agents absorbed through the tick cuticle, or systemically (via pour-on or injection), where they are ingested with blood by feeding ticks and exert their effects internally (Graf et al., 2004). However, repeated and widespread use of these compounds has led to the emergence of resistance in several tick species, including those of greatest economic importance in veterinary medicine (e.g., *Rhipicephalus* (*Boophilus*) *microplus*) (Rodriguez-Vivas et al., 2018). This escalating resistance underscores the urgent need for novel molecular targets and alternative tick control strategies.

Chitin, a linear polysaccharide composed of β-(1-4)-linked N-acetyl-D-glucosamine (GlcNAc) units, is a ubiquitous structural component of arthropods, fungi, and several other organisms. Notably, chitin and the enzymes responsible for its synthesis are absent in vertebrates, making them selective and biologically safe targets for disrupting arthropod physiology. In arthropods, chitin is embedded in protein matrices to form composite structures that provide mechanical strength, flexibility, and resilience (Muthukrishnan et al., 2012, Muthukrishnan et al., 2022). These chitin-containing structures include the cuticle, peritrophic matrix (PM) of the midgut, tracheae, and other specialized tissues. The cuticle serves as an exoskeleton that protects arthropods from physical damage, desiccation, and microbial invasion. Structurally, it is a multilayered composite with chitin localized in the procuticle, interspersed with proteins and, in some species, cross-linked with quinones to enhance rigidity. By comparison, the PM is a thinner, acellular chitin-protein layer that lines the midgut lumen and serves as a semi-permeable barrier, protecting the intestinal epithelium from mechanical abrasion and pathogen invasion while facilitating nutrient absorption and digestion.

Chitin synthesis is a tightly regulated biochemical process catalyzed by chitin synthases (CHSs), large multi-pass transmembrane glycosyltransferases that polymerize intracellular UDP-GlcNAc into chitin chains and extrude them into the extracellular space, where they are assembled into microfibrils (Yu et al., 2024). In insects, this process is spatially and functionally compartmentalized: *CHS1* (also known as *CHSA* or *krotzkopf verkehrt* (*KKV*) in *Drosophila*) operates at the apical membrane of epidermal cells to produce cuticular chitin, while *CHS2* (also known as *CHSB*) is localized to midgut epithelial cells and is responsible for chitin deposition into the PM (Merzendorfer, 2006). Coordinated regulation of chitin synthesis and turnover enables dynamic remodeling of chitinous structures and is essential for processes such as molting, tissue renewal, and gut function.

CHSs have long been implicated as targets of IGRs because of their essential roles in molting and cuticle formation, yet their precise molecular mode of action remained unresolved (Merzendorfer, 2006, Merzendorfer, 2013; Merzendorfer and Zimoch, 2003). Definitive genetic evidence emerged when etoxazole-resistant populations of the spider mite *Tetranychus urticae* were found to carry point mutations in *CHS1* within a conserved C-terminal transmembrane domain (Van Leeuwen et al., 2012). Subsequent CRISPR/Cas9-mediated introduction of homologous resistance mutations into the single *CHS1* gene of *Drosophila melanogaster* conferred high-level resistance to etoxazole, benzoylureas, and buprofezin, directly confirming CHS1 as the primary molecular target of these chemically diverse IGRs (Douris et al., 2016). Notably, resistance mutations affecting the same conserved residue have been independently identified in distantly related arthropods, including *Lepidoptera*, mites, and mosquitoes, underscoring the evolutionary conservation and genetic tractability of this drug-target interaction (Douris et al., 2016; Van Leeuwen et al., 2012).

Despite their presumed importance in tick physiology, the roles of CHSs remain largely unexplored. To address this gap, we performed a comprehensive analysis of *CHS* genes in *Ixodes ricinus*, an important European disease vector. We combined genomic and transcriptomic analyses with RNA interference (RNAi) and chemical inhibition to dissect CHS roles across key physiological contexts. By integrating molecular, structural, and functional approaches, we characterized *CHS* paralogs, mapped their tissue- and stage-specific expression, and defined their contributions to cuticle formation, PM biogenesis, tick feeding, development, and reproduction. Finally, we assessed whether tick-derived chitin influences the acquisition and transmission of *Borrelia*, a GlcNAc-auxotrophic pathogen.

## 2 Materials and methods

### 2.1 Ethics statement

All experiments were performed in accordance with the Animal Protection Act of the Czech Republic (No. 359/2012 Sb.) and Decree 501/2020 Sb. of the Ministry of Agriculture on the Protection of Experimental Animals, including relevant EU regulations, with the approval of the Ethics Committee of the Institute of Parasitology, Biology Centre, Ceske Budejovice, Czech Republic (Permits: 25/2020 and 5/2025).

### 2.2 Biological material

Adult ticks (*I. ricinus* females and males; males of this species do not feed and are included in feeding experiments to support female feeding) were collected in the České Budějovice district using the flagging method. Nymphs were obtained from the Institutional tick-rearing facility. Adult ticks (30 females with 30 males per guinea pig) were fed on guinea pigs and nymphs (20 nymphs per mouse) on BALB/c mice (both from local animal facility). The ticks were housed in glass boxes at 96% humidity, 24 °C with a 12 h light/12 h dark cycle. For both the RNAi and etoxazole treatments, we recorded feeding success, duration of feeding, and the weight of each tick after feeding. We also monitored tick survival, molting of nymphs, and larval hatching. The total weight of larvae hatched from each clutch was measured after freezing the larvae (-20 °C for 24 hours).

### 2.3 Database search and phylogenetic analysis

Tick *CHS* genes were identified in the NCBI database of the *Ixodes scapularis* genome (GCF_016920785.2) and *I. ricinus* transcripts using *Tribolium castaneum* CHS1 (NP_001034491) as a query sequence. The resulting genes and transcripts were translated into amino acid sequences (DNASTAR) and analyzed for domain structures using the NCBI CD search and for transmembrane helices using TOPCONS (https://topcons.cbr.su.se/). An unrooted phylogenetic tree was constructed using the Neighbor-Joining (NJ) method implemented in MEGA4 (https://www.megasoftware.net/), with bootstrap support based on 1,000 replicates. The analysis was performed on a multiple sequence alignment of the conserved Chitin_synt_C domain from arthropod CHS proteins generated using ClustalX. The full alignment is available at https://zenodo.org/records/18019462.

### 2.4 Nucleic acid extraction, cDNA synthesis, and quantitative real-time PCR

DNA and RNA were extracted with the NucleoSpin Tissue Kit (Macherey-Nagel) and the NucleoSpin RNA II Kit (Macherey-Nagel), respectively. RNA concentrations were measured with a NanoDrop ND-1000 spectrophotometer (Thermo Fisher Scientific) and RNA integrity was checked by agarose gel electrophoresis. cDNA synthesis was performed using the Transcriptor High Fidelity cDNA Synthesis Kit (Roche) with 0.5 μg RNA in a 20 μl reaction and anchored oligo(dT) primers. The cDNA was then diluted 20-fold in sterile water. The oligonucleotide primers for qRT-PCR (**Supplementary Table 1**) were designed with Primer3 (http://bioinfo.ut.ee/primer3-0.4.0/) and verified by PCR with Fast Start Master mix (Roche). Tick gene expression profiling (relative expression; biological triplicates) was performed as previously described (Hajdusek et al., 2016; Mahmood et al., 2020) using a QuantStudio 6 Flex Real-Time PCR System (Applied Biosystems; technical triplicates) and LightCycler 480 SYBR Green I Master chemistry (Roche). Melting curve analysis was performed for all SYBR assays to confirm the presence of a single specific amplification product. Results were normalized to *I. ricinus elongation factor 1* (*EF1*; GU074769) using the mathematical model of Pfaffl (Pfaffl, 2001).

### 2.5 RNA interference

DNA fragments of *CHS* genes were amplified from *I. ricinus* cDNA using gene-specific primers (**Supplementary Table 1**) containing *ApaI* and *XbaI* restriction sites and cloned into the pll10 vector with two T7 promoters in reverse orientation (Mahmood et al., 2020). The double-stranded RNA (dsRNA) was synthesized using the MEGASCRIPT T7 transcription kit (Ambion) as described previously (Hajdusek et al., 2009). As a control, green-fluorescent protein (GFP) dsRNA was synthesized from linearized plasmid pll6 (Levashina et al., 2001) under the same conditions. The dsRNA (3 μg/μl, nymph = 64 nl, adult tick = 345 nl) was injected into the hemocoel (through the coxa III) of the unfed tick *I. ricinus* using the microinjector (Drummond). After inoculation, the ticks were allowed to rest in a humid chamber for three days and then fed on mice (three mice per group, 20 nymphs per host) or guinea pigs (one guinea pig per group, 30 adult females (with 30 untreated males) per host). The level of gene silencing was verified by quantitative real-time PCR (qRT-PCR) in pools of five fully-fed nymphs (five biological replicates) and compared with the dsGFP control group as described previously (Mahmood et al., 2020).

### 2.6 *Borrelia* transmission model

Transmission of *Borrelia afzelii* CB43 from the dsRNA-treated nymphs to the mice was performed as described previously (Honig Mondekova et al., 2017; Pospisilova et al., 2019; Trentelman et al., 2020). Briefly, ten dsRNA-injected *B. afzelii*-infected nymphs of *I. ricinus* were fed on six-week-old C3H/HeN mice (AnLab, five mice per group). Infection of the mice was checked weekly by PCR amplification of the *flagellin* gene. Four weeks after tick detachment, the mice were sacrificed and the presence of *Borrelia* in ear, bladder, and heart tissues was tested by PCR. All experiments with *Borrelia* were performed under BSL2 conditions.

Sera from mice were analyzed by the enzyme-linked immunosorbent assay (ELISA) four weeks after feeding of infected nymphs to determine antibody responses against *B. afzelii* CB43 infection. Spirochetes were cultured in BSK-H medium at 33 °C for one week to a concentration of approximately 1×10^7^ spirochetes/ml. The cultures were centrifuged at 2,500 g for 10 minutes at room temperature, and the resulting pellet was resuspended in an equal volume of PBS. The suspension was sonicated five times for 30 seconds each and then centrifuged again (2,500 g, 10 minutes, room temperature). The supernatant containing the *Borrelia* lysate was stored at -20 °C until use. 96-well plates were coated with 0.5 µg *Borrelia* lysate in 50 µl coating buffer (15 mM Na_2_CO_3_, 35 mM NaHCO_3_, pH 9.6) per well and incubated overnight at 4 °C. The next day, the coating buffer was discarded and the plates were blocked with 10% fetal bovine serum (Sigma) in PBS for 45 minutes at 37 °C in a humid chamber. After blocking, the wells were washed three times with 0.05% PBS-T. Primary mouse sera were added in twofold serial dilutions in the blocking solution (starting at 1:100) and plates were incubated at 37 °C for 45 minutes. The wells were then washed three times with PBS-T and incubated for 45 minutes at 37 °C with rabbit anti-mouse IgG secondary antibody (1:2,000, Sigma, (A9044)). After incubation, the plates were washed three times with PBS-T. The reaction was developed by adding a substrate buffer consisting of 0.2 M Na_2_HPO_4_ and 0.1 M citric acid (pH 5.0), supplemented with 2 mg *o*-phenylenediamine dichloride (Sigma) and 2 µl 30% H_2_O_2_ in 5 ml substrate buffer. After color development, the reaction was stopped by addition of 50 µl of 2M H_2_SO_4_ and the absorbance was measured at 490 nm using a Tecan (Schoeller) plate reader. The cut-off value for seropositivity was determined as an optical density value that was at least twice as high as the corresponding absorbance of the pre-immune sera at the same dilution.

### 2.7 *Borrelia* acquisition model

*Borrelia* acquisition was performed as previously described (Jalovecka et al., 2024). Briefly, six-week-old C3H/HeN mice (AnLab) were infected by subcutaneous injection of 1x10^7^ *B. afzelii* CB43. Four weeks later, twenty dsRNA-injected nymphs were fed on each mouse (six mice per group) until repletion. Whole-body spirochete numbers were quantified in the nymphs by qRT-PCR (*flagellin*) immediately after feeding and at one, two, and three weeks after feeding (24 nymphs per time point, equal numbers of females and males) as previously described (Pospisilova et al., 2019).

### 2.8 Topical application of etoxazole on ticks

Etoxazole (Cayman chemical (25782)) was dissolved in 100% ethanol to obtain a 1 mM solution, which was then filtered through a 0.22 µm syringe filter. A 31.6 µM solution was prepared by diluting the stock in 100% ethanol. Nymphs were immobilized ventrally on adhesive tape, and 32 nl of etoxazole (1 mM or 31.6 µM) was topically applied as a droplet to the dorsal cuticle posterior to the scutum using a microinjector (Drummond). Control ticks received 100% ethanol. After application, ticks were incubated at room temperature for 15 minutes, gently dried with filter paper, detached from the tape, and placed in a humid chamber for 24 hours to recover. Ticks were then fed on BALB/c mice (20 nymphs per mouse, three mice per group), and both feeding and subsequent molting into adults were monitored.

### 2.9 Membrane feeding of ticks

Membrane feeding of ticks was performed in a six-well plate format according to the protocol of Kröber and Guerin (Kröber and Guerin, 2007) with the previously described modifications (Perner et al., 2016, Perner et al., 2021; Šíma et al., 2024). Each feeding unit was equipped with a thin silicone membrane (~80 µm thickness). Whole blood from cattle was obtained from a local abattoir and manually defibrinated. Gentamicin (final concentration of 10 µg/ml, Sigma (G1264)) and Nystatin (final concentration of 10 U/ml, Sigma (N1638)) were added to the blood. Etoxazole (Cayman chemical (25782)) was dissolved in 100% ethanol to obtain a 100 mM stock solution, which was then filtered through a 0.22 µm syringe filter. A concentration series was prepared by diluting this stock solution in 100% ethanol. For each feeding condition, 31 µl of the appropriate dilution (or 100% ethanol as a control) was mixed with 3.1 ml of blood (a 100-fold dilution) in a six-well plate and presented to the ticks.

Adult females (12 females, together with 10 males to support feeding) and 25 nymphs (both from the Institutional tick-rearing facility) were initially placed in each feeding unit with unspiked blood for two and one days, respectively, to ensure proper attachment of the ticks to the membrane. The unspiked blood was then replaced with blood spiked with etoxazole at various concentrations and fresh gentamicin (final concentration of 10 µg/ml) and refreshed every 12 hours until the end of feeding. Ethanol, used as a solvent for etoxazole, served as a negative control with a final ethanol concentration of 1% in the blood, which corresponded to the ethanol concentration in each well with etoxazole. Fully-engorged ticks were collected after they had spontaneously detached from the silicone membrane, whereupon feeding success and post-feeding survival were assessed.

### 2.10 Light and transmission electron microscopy

The midguts and cuticles from fully-fed nymphs (for cuticles one week after detachment) were dissected and fixed overnight at 4 °C in 2.5% glutaraldehyde, washed in Na-phosphate buffer (pH 7.2), and incubated with 2% osmium tetroxide. Subsequently, the samples were dehydrated in ascending acetone series and infiltrated stepwise in acetone mixed with EMbed 812 Epon resin. Finally, the embedded samples were polymerized at 62 °C for 48 hours. The semi-thin sections (~400 nm) of cuticle samples were stained with Toluidine blue for 1 minute at 60 °C and examined using the BX 83 microscope equipped with a DP 70 camera (Olympus). Endocuticle thickness was measured using ImageJ software. Ultra-thin sections of midguts and cuticle (~90 nm) were contrasted in uranyl acetate for 30 minutes and in lead citrate for 20 minutes. The transmission electron microscope (TEM) JEM 1400 Flash (JEOL) equipped with a XAROSA camera (SIS) was used for sample investigation.

### 2.11 Immunogold labeling of tick tissues

Midguts were dissected from fully-fed nymphs and fixed in 4% formaldehyde with 0.1% glutaraldehyde in 0.1 M HEPES buffer (pH 7.2) for 1 hour at room temperature. The samples were then washed in HEPES buffer and cryopreserved in 2.3 M sucrose for 48-72 hours at 4 °C before freezing in liquid nitrogen. Ultrathin cryosections (~90 nm) were cut at -100 °C using a LEICA EM UC-6/FC-6 cryo-ultramicrotome, collected using a drop of 1.15 M sucrose/1% methylcellulose solution (25 cp, Sigma), and transferred onto Formvar carbon-coated grids, and stored at 4 °C. Prior to labelling, grids were incubated for 5 minutes at 37 °C in HEPES buffer. Grids were optionally treated with Proteinase K (Macherey-Nagel (740506), ~50 U/ml, diluted 1:20) at 37 °C for 1 hour, followed by three washes in blocking solution, each lasting 15 minutes. All grids were blocked for 1 hour at room temperature in 3% BSA in HEPES buffer. Labeling was performed using biotinylated Wheat germ agglutinin (WGA, Vector Laboratories, B-1025, 1:200 in 1% BSA/HEPES buffer from stock solution of 2.5 mg/ml) for 20 minutes. Control sections were treated with WGA preincubated with chitin hydrolysate (Vector Laboratories, SP-0090). After washing in 1% BSA, the sections were incubated for 1 hour at room temperature with streptavidin conjugated to 5 nm gold nanoparticles (BBI Solutions, 019970) diluted 1:40 in 1% BSA. The sections were then washed in 0.1 M HEPES buffer, postfixed in 1% glutaraldehyde in HEPES buffer for 5 minutes, rinsed in demineralized H_2_O and finally contrasted in uranyl acetate for 15 minutes and in lead citrate for 10 minutes. The sections were carbon coated. At least 10 sampled areas per section were analyzed across three individual ticks per group collected from the same feeding experiment.

The cuticles of the fully-fed nymphs were fixed in a solution of 4% formaldehyde and 0.1% glutaraldehyde in 0.1 M HEPES buffer at 4 °C overnight. The samples were then washed three times for 10 minutes in wash buffer (4% glucose in 0.1 M sodium phosphate buffer, pH 7.3) and dehydrated with ascending ethanol dilutions. The dehydrated samples were infiltrated with LR White resin (London Resin Company, Ltd.) at resin to 100% ethanol ratios of 2:1, 1:1, and 1:2 (each for 1.5 hours at room temperature), followed by overnight incubation at room temperature in pure LR White resin. The samples were then transferred to resin-filled capsules and polymerized at 50 °C for 24 hours. Ultrathin sections (~90 nm) were cut and placed on nickel grids. All grids were blocked for 1 hour at room temperature in 3% bovine serum albumin (BSA) in HEPES buffer. Labeling with WGA was performed as described above. After washing twice (5 minutes each) in 1% BSA, the sections were incubated for 1 hour at room temperature with streptavidin conjugated with 5 nm gold nanoparticles (BBI Solutions, 019970) diluted 1:40 in 1% BSA. Control sections were treated with streptavidin only. Sections were then washed in 0.1 M HEPES buffer, postfixed in 1% glutaraldehyde in HEPES buffer for 5 minutes, rinsed in demineralized H_2_O, and finally contrasted with uranyl acetate for 15 minutes followed by lead citrate for 10 minutes. At least 10 sampled areas per section were analyzed across three individual ticks per group collected from the same feeding experiment.

### 2.12 Quantification of etoxazole in blood by liquid chromatography-tandem mass spectrometry

The method was adapted from a previous protocol for the quantitative analysis of fipronil and ivermectin (Šíma et al., 2024). Briefly, 800 µl of methanol was added immediately after removal of each sample from the thermostat for protein precipitation. Samples were briefly shaken by hand and then placed in an ultrasonic bath at 0 °C for 5 minutes. They were then centrifuged (4,650 g for 5 minutes at 4 °C) and a 200 µl aliquot of the supernatant was collected for HPLC-MS/MS analysis. Quantitative analysis of etoxazole was performed in technical triplicates using a liquid chromatography system (Accela 600 pump and Accela AS autosampler) coupled to an LTQ-XL mass spectrometer (Thermo Fisher Scientific). Chromatographic separation was performed on a Zorbax Eclipse Plus C18-Rapid Resolution HD column (50 × 3 mm ID, 1.8 µm (Agilent Technologies), maintained at 35 °C. Five µl of each sample was injected. The mobile phase consisted of solvent A) 5 mM ammonium formate in methanol and solvent B) 5 mM ammonium formate in water, added at a flow rate of 400 µl/minute under the following gradient conditions: 0 minutes, 20:80 (A:B); 4.5 minutes, 100:0; 8 minutes, 100:0; 8.1 minutes, 20:80; 10 minutes, 20:80. The mass spectrometer was operated in positive ion detection mode (2.5 kV) with capillary and source heating temperatures of 300 °C each, sheath gas at 35 AU, auxiliary gas at 10 AU, and sweep gas at 1 AU. The eluted ions were detected in full scan mode (200-1,000 Da) and in MS/MS mode. For the MS/MS analysis of etoxazole, the ion at 360.4 Da [M+H]^+^ (NCE 22, 4 m/z) was used (**Supplementary Figure 1**). Data acquisition and processing was performed using XCalibur 4.0 software (Thermo Fisher Scientific).

### 2.13 Hydrolysis of tick cuticles and analysis of chitin-derived glucosamine by liquid chromatography-mass spectrometry

Dorsal cuticle from three fully-fed nymphs one week after detachment were pooled per sample (five biological replicates per group) and weighed into reaction vials (3.5-5.5 mg). To each vial, 0.5 ml of 8 M hydrochloric acid (HCl) was added. Hydrolysis was performed at 100 °C for 2 hours in a dry oven. After hydrolysis, the samples were cooled to room temperature overnight and then evaporated to dryness using a SpeedVac concentrator (RVC 2-25 CDplus, Martin Christ, Osterode am Harz, Germany). The dried residues were reconstituted in 250 µl deionized water, vortexed for 1 minute, and sonicated for 5 minutes in an ice-cold water bath. The mixture was transferred to a new vial. The reaction vial was then rinsed with another 250 µl of deionized water, vortexed, and sonicated again. The two extracts were pooled. The samples were centrifuged at 4,500 g for 10 minutes at 4 °C. A 480 µl aliquot of the supernatant was transferred to conical LC/MS vials and evaporated to dryness in a SpeedVac. The dried residues were reconstituted in 24 µl of 50% (v/v) acetonitrile by successively adding 12 µl of water and 12 µl of acetonitrile. This resulted in a 20-fold concentration compared to the original supernatant. LC/MS analysis was performed as previously described (Moos et al., 2022). Glucosamine (GlcN) was analyzed using a Q Exactive Plus Orbitrap mass spectrometer coupled to a Dionex Ultimate 3000 UHPLC system with open autosampler (Thermo Fisher Scientific, San Jose, CA, USA). The instrument operated in electrospray ionization (ESI) mode with positive (PESI) ion detection. The full MS scan mode was used over a mass range of 75-1000 m/z. GlcN was identified based on the authentic standard (Merck (G4875)) with the following parameters: (RT) 9.9 min, [M+H]^+^ 180.0867. The instrument parameters were: resolving power 70,000; AGC target 3 × 10^6^; maximum injection time 100 ms. The settings of the ESI source were as follows: spray voltage ±3,000 V; capillary temperature 350 °C; probe temperature 350 °C; sheath gas flow 60 au; auxiliary gas 20 au; substitute gas 1 au; S-lens RF level 60 au. The external lock mass m/z 622.0290 (hexakis(2,2-difluoroethoxy)phosphazene) was used for internal calibration. Chromatographic separation was performed using a SeQuant ZIC-pHILIC column (150 mm × 4.6 mm i.d., 5 μm particle size; Merck KGaA, Darmstadt, Germany) maintained at 35 °C. The mobile phase consisted of solvent A) acetonitrile and solvent B) 20 mM ammonium carbonate, pH 9.2, adjusted with NH_4_OH. The flow rate was 450 µl/minute, the injection volume 5 µl. The elution gradient was as follows: 0 minutes, 20% B; 20.0 minutes, 80% B; 20.1 minutes, 95% B; 23.3 minutes, 95% B; 23.4 minutes, 20% B; 30.0 minutes, 20% B. The data were recorded and processed using MetaboliteMapper and Xcalibur software version 4.0 (Thermo Fisher Scientific). GlcN peak areas were normalized to a stable endogenous amino acid pool to control for technical variability. Peak areas for twenty abundant amino acids (tyrosine, valine, proline, D/L-glutamate, asparagine, 4-aminobutyrate, aspartate, glycine, cystine, phenylalanine, leucine, methionine, threonine, alanine, β-alanine, histidine, serine, ornithine, lysine, and arginine) were extracted from the same LC-MS datasets. These amino acids showed no treatment-dependent changes and were used as the internal reference pool. The sum of their peak areas was used as the normalization factor for GlcN (**Supplementary Table 2**). Raw data for this analysis, as well as for the preceding method (quantification of etoxazole in blood by LC-MS/MS), are deposited here (https://zenodo.org/records/18019462).

### 2.14 Statistical analyses

Statistical significance was assessed using GraphPad Prism 10.0 (GraphPad Software, San Diego, CA, USA). Differences between two groups were assessed using the nonparametric Mann-Whitney U test. For comparisons involving multiple groups, the Kruskal-Wallis test was applied followed by Dunn’s multiple comparisons test. Adjusted *P* values were used to determine statistical significance, with *P* ≤ 0.05 considered significant. Nonparametric tests were chosen to provide robust comparisons without assuming normality, particularly given the small sample sizes. Where sample sizes allowed, data distributions were inspected. However, formal assessment of distribution shape was not feasible for small groups.

## 3 Results

### 3.1 The tick *I. ricinus* possesses three *CHS* genes

**Figure 1:**
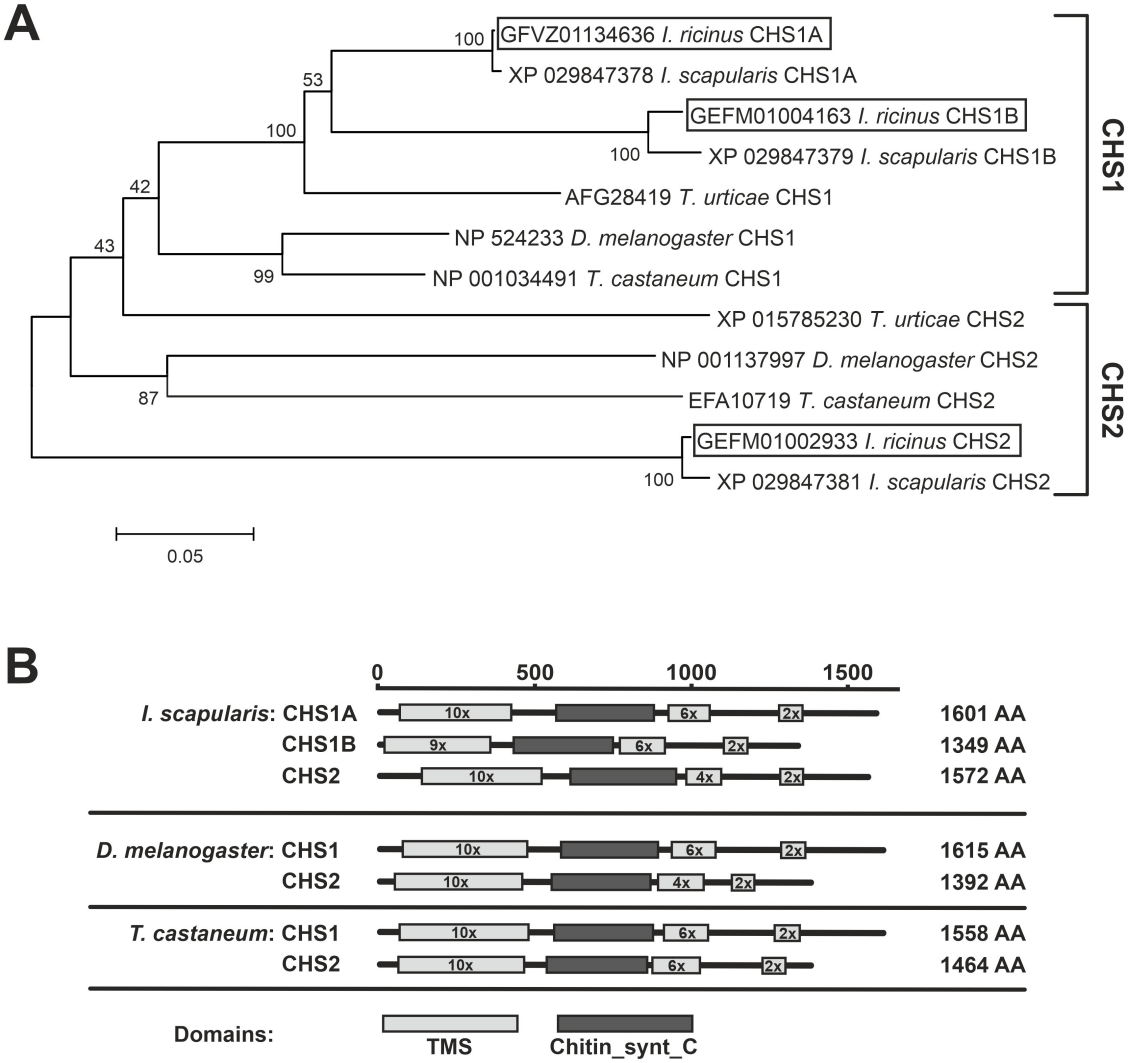
*Ixodes* ticks possess three types of CHSs. **(A)** Phylogenetic tree of selected arthropod CHSs. The unrooted neighbor-joining tree based on a multiple sequence alignment of the conserved Chitin_synt_C domain from arthropod CHS proteins. *I. ricinus* sequences are enclosed in boxes. Numbers at the nodes indicate bootstrap support values; scale bar = 0.05 substitutions per site. Ticks: *Ixodes ricinus*, *Ixodes scapularis*; mite: *Tetranychus urticae*; insects: *Tribolium castaneum*, *Drosophila melanogaster*. **(B)** Domain structures of tick and insect CHSs as predicted by TOPCONS. TMS, transmembrane spans.

To investigate the presence and diversity of *CHS* genes in *Ixodes* ticks, we performed a comprehensive search of the *I. scapularis* genome (a species closely related to *I. ricinus* with high-quality genomic data (De et al., 2023)) as well as *I. ricinus* transcriptomic datasets available in the NCBI database. This analysis revealed three different *CHS* genes in the genome of *I. scapularis*. All three *CHS* genes are located within a single genomic locus (NW_024609837.1:10408831-10630528), positioned consecutively in the genome, with only one unrelated gene, a *high-affinity choline transporter 1* (XM_042289136.1), inserted between the *CHS1A* and *CHS1B* genes. Subsequently, homologs of these genes were identified in the transcriptomes of *I. ricinus*. The phylogenetic analysis clustered the *Ixodes* CHSs into three groups: CHS1A, CHS1B, and CHS2 (**Figure 1A**). *CHS1A* was represented in all databases. In contrast, *CHS1B* was only identified in the genome of *I. scapularis* and in a single transcriptomic BioProject (PRJNA311553) of *I. ricinus*. Because all other tick CHSs, as well as CHSs from *Drosophila* and *Tribolium*, contain ten predicted transmembrane spans (TMS) N-terminal to the catalytic domain (**Figure 1B**), it is likely that the start codon of CHS1B, which contains only nine such domains, is mispredicted and that its N-terminal region is thus missing from the current annotation. The *CHS2* genomic locus in *I. scapularis* encodes a single *CHS* gene with four annotated transcript isoforms (XM_040222999.3, XM_040222998.1, XM_042288818.1, and XM_029991521.2).

The overall domain architecture of *Ixodes* CHSs is consistent with other arthropod orthologs, consisting of an N-terminal multipass membrane region, a large cytosolic catalytic domain, and a C-terminal transmembrane block (called the extrusion domain) that mediates chitin chain translocation through the membrane (**Figure 1B**). In insects, the major loop of the extrusion domain, located between the 6-TMS block and the final 2-TMS region, was predicted to be on the extracellular side (Muthukrishnan et al., 2012). However, our analysis of tick and insect CHSs using TOPCONS predicted this loop to face the cytoplasmic side. This revised topology is consistent with recent cryo-EM structures of fungal CHS enzymes, in which the catalytic domain and all major regulatory loops are strictly cytoplasmic, whereas the extracellular side is limited to short loops between individual TMS that form the membrane-embedded exit pore (Chen et al., 2022).

### 3.2 Three *CHS* genes show different tissue and developmental expression profiles

**Figure 2:**
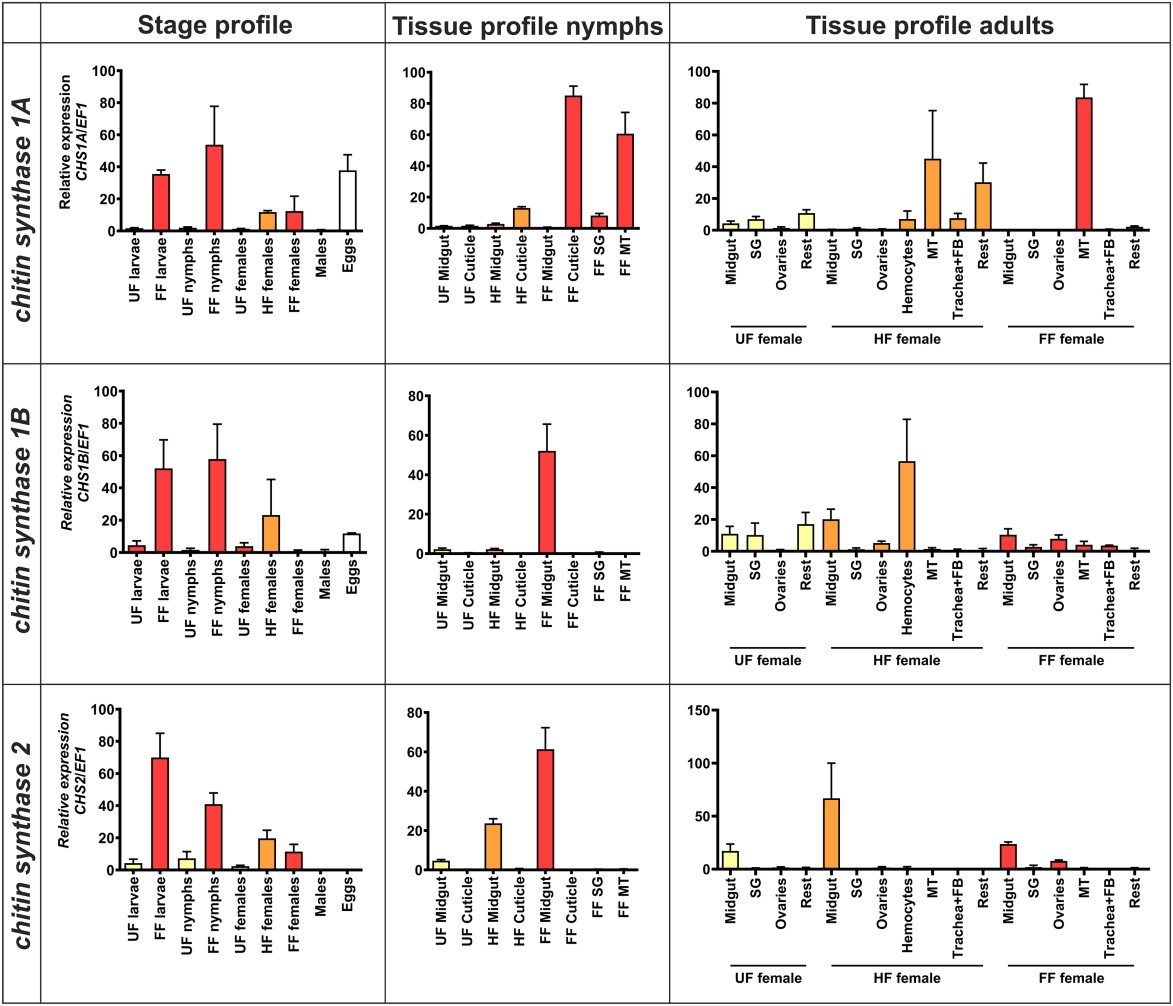
Tissue and developmental stage-specific expression profiles of *I. ricinus CHS genes*. Relative expression (qRT-PCR) of tick *CHS* genes in different tick developmental stages and tissues of adult ticks and nymphs. The expression was normalized to tick *elongation factor 1* (*EF1*). Results represent the mean of three independent biological replicates. The highest individual value for a given gene in each panel was set to 100% and all other values were expressed relative to this value. UF, Unfed; HF, half-fed; FF, fully-fed; SG, salivary glands; MT, Malpighian tubules; FB, fat body.

To determine the tissue- and stage-specific expression patterns of the *CHS* genes in ticks, we performed qRT-PCR profiling at different developmental stages (larvae, nymphs, and adults) before and after blood feeding, and from tissues of unfed, half-fed (24 hours for nymphs, five days for adult females), and fully-fed nymphs and adult females. All three *CHS* genes showed low expression in the unfed stages (**Figure 2**). However, their expression was strongly upregulated by blood feeding. This upregulation was more pronounced in the immature tick stages (larvae and nymphs) than in the adult females. In developing embryos (eggs collected six weeks after oviposition), we detected expression of *CHS1A* and *CHS1B*.

At the nymphal stage, *CHS1A* showed high and feeding-dependent expression in the cuticle (epithelial cells). The gene was also well expressed in the Malpighian tubules of the fully-fed nymphs. In feeding adult females, *CHS1A* was mainly expressed in the rest of the body (which consists mainly of cuticle) and in the Malpighian tubules. In the fully-fed adult ticks, expression was exclusively restricted to the Malpighian tubules. *CHS1B* was expressed exclusively in the midgut of fully-fed nymphs. In adults, it was detected in most tissues of unfed individuals (including the cuticle and midgut), was strongly expressed in the hemocytes of half-fed ticks and showed relatively high abundance in the midgut and ovaries of fully-fed females. Finally, *CHS2* showed an increasing expression pattern in the nymphs, which was exclusively restricted to the midgut tissue, reaching a maximum in the fully-fed nymphs. In adult females, *CHS2* was expressed in the midgut of ticks before, during, and after feeding, with notable expression in the ovaries of fully-fed adults. In summary, the distinct tissue-specific expression profiles of the three tick *CHS* genes suggest that they play specific roles during blood feeding and development.

### 3.3 RNAi shows that CHS1A is essential for chitin synthesis in the cuticle

**Figure 3:**
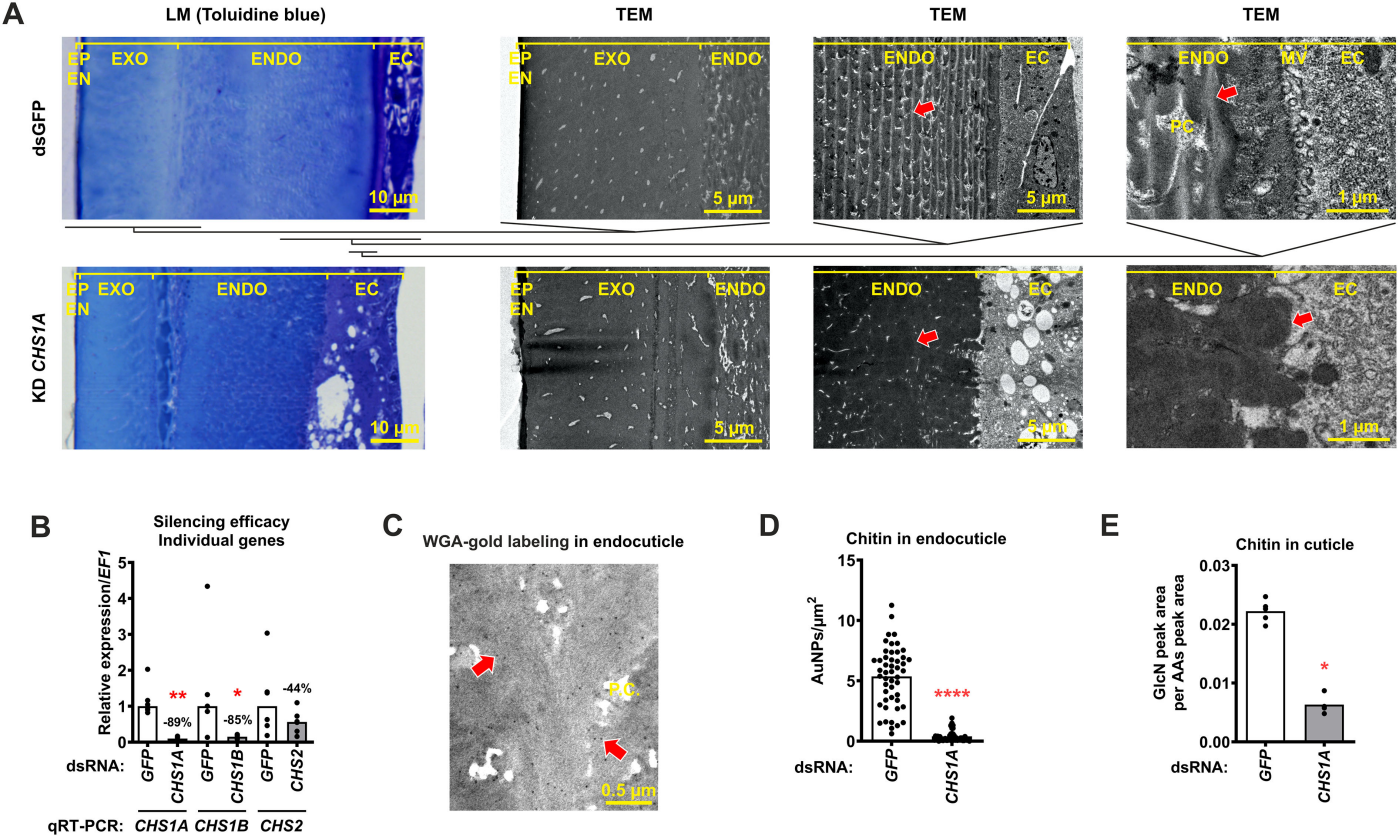
Silencing of the *CHS1A* gene by RNAi alters the morphology of the tick cuticle. **(A)** Representative microscopy images showing the structure of the tick cuticle in dsGFP control nymphs and *CHS1A*-KD nymphs one week after feeding. Light microscopy (LM), transmission electron microscopy (TEM), epithelial cells (EC), endocuticle (ENDO), exocuticle (EXO), epicuticle (EP), envelope (EN), microvilli (MV), pore canals (canaliculi, PC). Red arrows show the lamellar structure of the endocuticle in the dsGFP control compared to the disrupted endocuticle in the *CHS1A*-KD group. The horizontal bars beneath the dsGFP LM image indicate the cuticle regions selected for subsequent TEM imaging; comparable anatomical regions were consistently selected for TEM across all groups. **(B)** Efficacy of gene silencing in fully-fed nymphs (whole bodies) measured by qRT-PCR. Each dot represents one pool of five nymphs (five biological replicates), and medians are plotted. **(C)** Representative TEM image of WGA-gold labeling (red arrows) in the endocuticle of dsGFP control nymphs, illustrating the labeling signal quantified in (D). **(D)** Labeling density (LD) of WGA staining (gold particles/µm² cuticle) for the dsGFP control and *CHS1A*-KD groups. Each dot represents the LD value of at least 10 sampled areas per section, across three individual ticks per group collected from the same feeding experiment. Gold nanoparticles (AuNPs). **(E)** Relative amounts of glucosamine (GlcN; the end product of chitin hydrolysis) in the cuticles of fully-fed nymphs quantified by liquid chromatography-mass spectrometry (LC-MS) and normalized to the amino acid (AA) content of each sample. *P ≤ 0.05; **P ≤ 0.01; ****P ≤ 0.0001.

The tick cuticle, which is synthesized by the underlying epithelial cells (epidermis), consists of the procuticle (divided into endocuticle and exocuticle), the epicuticle, and the envelope (in ascending order from the epidermis) (Starck et al., 2018). It is generally assumed that the chitin secreted by the epithelial cells is exclusively present in the procuticle (Muthukrishnan et al., 2022). To investigate the specific role of individual *CHS* genes in cuticle synthesis during and after feeding, we performed RNAi-mediated gene knockdowns (KDs) by injecting gene-specific dsRNA into nymphs and examined the resulting cuticle phenotypes by light and electron transmission microscopy one week after feeding. As previously reported, dsRNA delivery via the hemolymph triggers systemic gene silencing across multiple tissues and persists into later developmental stages (Hajdusek et al., 2009; Jalovecka et al., 2024). In agreement with this, qRT-PCR analysis of fully-fed nymphs confirmed downregulation of the targeted *CHS* transcripts following KD (**Figure 3B**). In the *CHS1A*-KD group alone, the thickness of the endocuticle, a layer newly synthesized after tick attachment (Starck et al., 2018), was significantly reduced due to its altered morphology (**Figure 3A**; **Supplementary Figures 2**, **3A**). In contrast to the dsGFP control in which the endocuticle remained intact and exhibited a layered, lamellar structure, the *CHS1A*-KD group exhibited a disrupted endocuticle composed of amorphous material. In addition, no pore canals (canaliculi) formed in the KD group compared to the control group. The structure of the exocuticle remained unchanged in all groups. The *CHS1A*-KD ticks also appeared more fragile at dissection and frequently ruptured, indicating impaired cuticle integrity.

To confirm that chitin content was reduced in the cuticle of *CHS1A*-KD ticks, we quantified chitin using immunogold electron microscopy and staining with WGA lectin, a specific marker for chitin-rich structures (GlcNAc). The gold particle signal (**Figure 3C**) was more pronounced in the endocuticle than in the exocuticle of the dsGFP control ticks (**Supplementary Figure 4A**). Its abundance was significantly reduced in the cuticle of *CHS1A*-KD ticks (**Figure 3D**). In addition, we quantified the amount of GlcN released from the cuticle chitin by acid hydrolysis and found that the GlcN content was also significantly lower in the *CHS1A*-KD group (**Figure 3E**). Taken together, these two analyses confirmed a significant reduction in cuticle chitin content in *CHS1A*-KD ticks and identified *CHS1A* as the key enzyme responsible for chitin synthesis in the tick cuticle.

### 3.4 All three CHSs are required for chitin deposition in the PM

**Figure 4:**
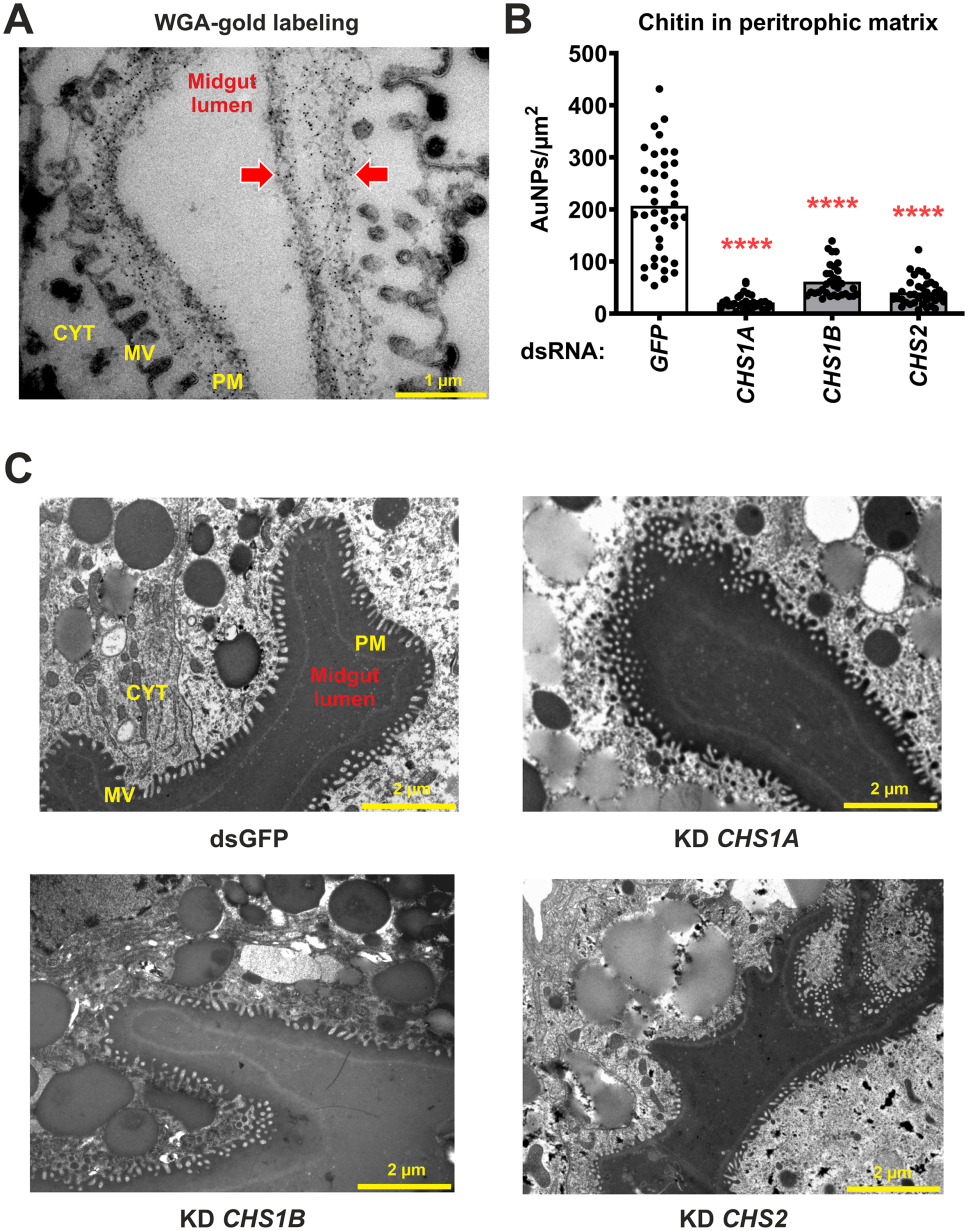
Silencing of the individual *CHS* genes by RNAi reduces the chitin content in the PM of the midgut. **(A)** Representative TEM image showing the chitin content in the PM of the midgut of a fully-fed dsGFP control nymph labeled with WGA-gold (red arrows). Cytoplasm (CYT), microvilli (MV), peritrophic matrix (PM), wheat germ agglutinin (WGA). Representative images for the individual *CHS* KD groups are shown in **Supplementary Figure 5**. **(B)** Labeling density (LD) of WGA staining (gold particles/µm² PM) for each KD group. Each dot represents the LD value of at least 10 sampled areas per section, across three individual ticks per group collected from the same feeding experiment. Gold nanoparticles (AuNPs). **(C)** Representative TEM images of tick midgut sections for each KD group. ****P ≤ 0.0001.

By silencing *CHS* genes through RNAi, we also investigated the specific role of individual CHSs in the midgut of *I. ricinus* nymphs. The chitin content in the midgut PM was detected and quantified by immunogold electron microscopy and staining with WGA lectin. To enhance the accessibility of chitin for WGA, the midgut sections were pretreated with Proteinase K to remove proteins that could hinder access to GlcNAc residues. As a result, chitin labeling was detected along the entire PM in the dsGFP control group, from the luminal surface adjacent to the gut contents to the tips of the midgut microvilli (**Figure 4A**). In contrast to the cuticle, where only KD of *CHS1A* affected chitin levels, KD of each individual *CHS* gene resulted in a significant decrease in chitin content in the PM (**Figure 4B**; **Supplementary Figure 5**). To verify that *CHS1A* dsRNA does not indirectly alter the expression of non-target, midgut-specific paralogs, we quantified *CHS1B* and *CHS2* transcripts in the dsGFP control and *CHS1A*-KD groups by qRT-PCR in fully-fed nymphs (**Supplementary Figure 6**). *CHS1A* KD was specific and did not affect *CHS1B* or *CHS2* expression. Despite the reduced chitin content, the overall presence and thickness of the PM, as revealed by electron microscopy, remained comparable between the individual KDs and the dsGFP control (**Figure 4C**). In summary, our results demonstrate that chitin is a structural component of the tick PM and that all three tick CHSs contribute to its synthesis.

### 3.5 Silencing of *CHS* genes by RNAi impairs tick feeding and development

**Figure 5:**
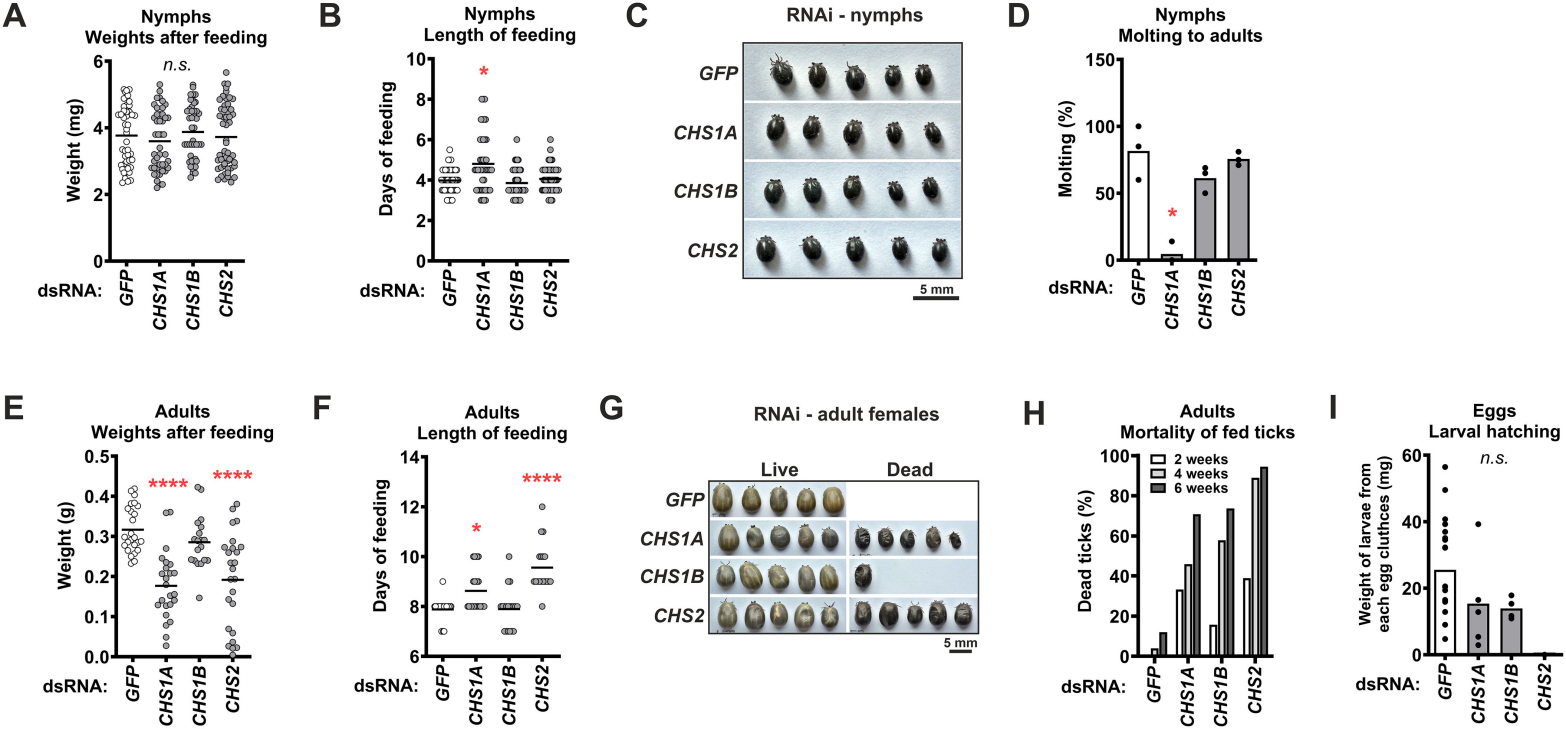
Silencing of *CHS* genes by RNAi reduces feeding and development of ticks. **(A)** Weight of fully-fed nymphs pre-injected with dsRNA. Results are from a single experiment, with each condition tested on three independent hosts (3 mice per group, 20 nymphs per mouse). **(B)** Duration of nymph feeding. **(C)** Representative images of fully-fed nymphs. **(D)** Success of molting of fed nymphs to adults. **(E)** Weight of fully-fed adult females pre-injected with dsRNA. Results are from a single experiment, with each condition tested on one host (1 guinea pig per group, 30 adult females per host, with 30 males to support feeding). **(F)** Duration of feeding of adult females. **(G)** Representative images of fully-fed adult females. **(H)** Cumulative mortality of adult females at 2, 4, and 6 weeks after feeding. **(I)** Total weight of all larvae hatched from individual egg clutches. Each dot represents one clutch. *P ≤ 0.05; ****P ≤ 0.0001; *n.s.*, not significant *P* ≥ 0.05.

To investigate the importance of CHSs for tick physiology, we fed RNAi-silenced nymphs and adults of *I. ricinus* (the vast majority of ticks survived dsRNA injection until feeding) on mice and guinea pigs, respectively. We investigated feeding success, post-feeding weight, mortality during or after feeding, molting of nymphs to adults, and adult oviposition together with subsequent larval hatching. We observed that silencing the *CHS* genes in the nymphs had no significant effect on feeding success or the weight and size of engorged ticks (**Figures 5A, C**). However, the duration of feeding was slightly, albeit significantly, prolonged in the *CHS1A-*KD group (**Figure 5B**). Importantly, in this group, molting from nymphs to adults was almost completely blocked (only 2 of 40 nymphs molted), with many nymphs darkening and desiccating (**Figure 5D**).

The effect of *CHS* genes silencing was more pronounced in adult ticks. The silencing of *CHS1A* and *CHS2* led to a significantly prolonged feeding time and a lower weight of fully-fed females (**Figures 5E, F**). In addition, the shape of the *CHS1A*-KD fully-fed ticks was irregular compared to the dsGFP control (**Figure 5G**). In the post-feeding period, the cumulative mortality rate was higher in all three KD groups than in the control group, with *CHS2* KD showing the highest mortality of up to 94% within six weeks after feeding (**Figure 5H**). Among the surviving ticks (7 of 25 in the *CHS1A*-KD group and 5 of 19 in the *CHS1B*-KD group), egg laying occurred, but the number of larvae hatching from these eggs was reduced by 40% and 45%, respectively, as measured by the total weight of larvae per clutch and compared with the dsGFP control (**Figure 5I**). In the *CHS2*-KD group, only 1 of 25 fed females laid eggs, but none of these eggs hatched. In summary, RNAi-mediated silencing of *CHS* genes had a profound effect on tick feeding, survival, and subsequent development, highlighting the critical role of CHSs in tick physiology.

### 3.6 Etoxazole impairs tick feeding and development

**Figure 6:**
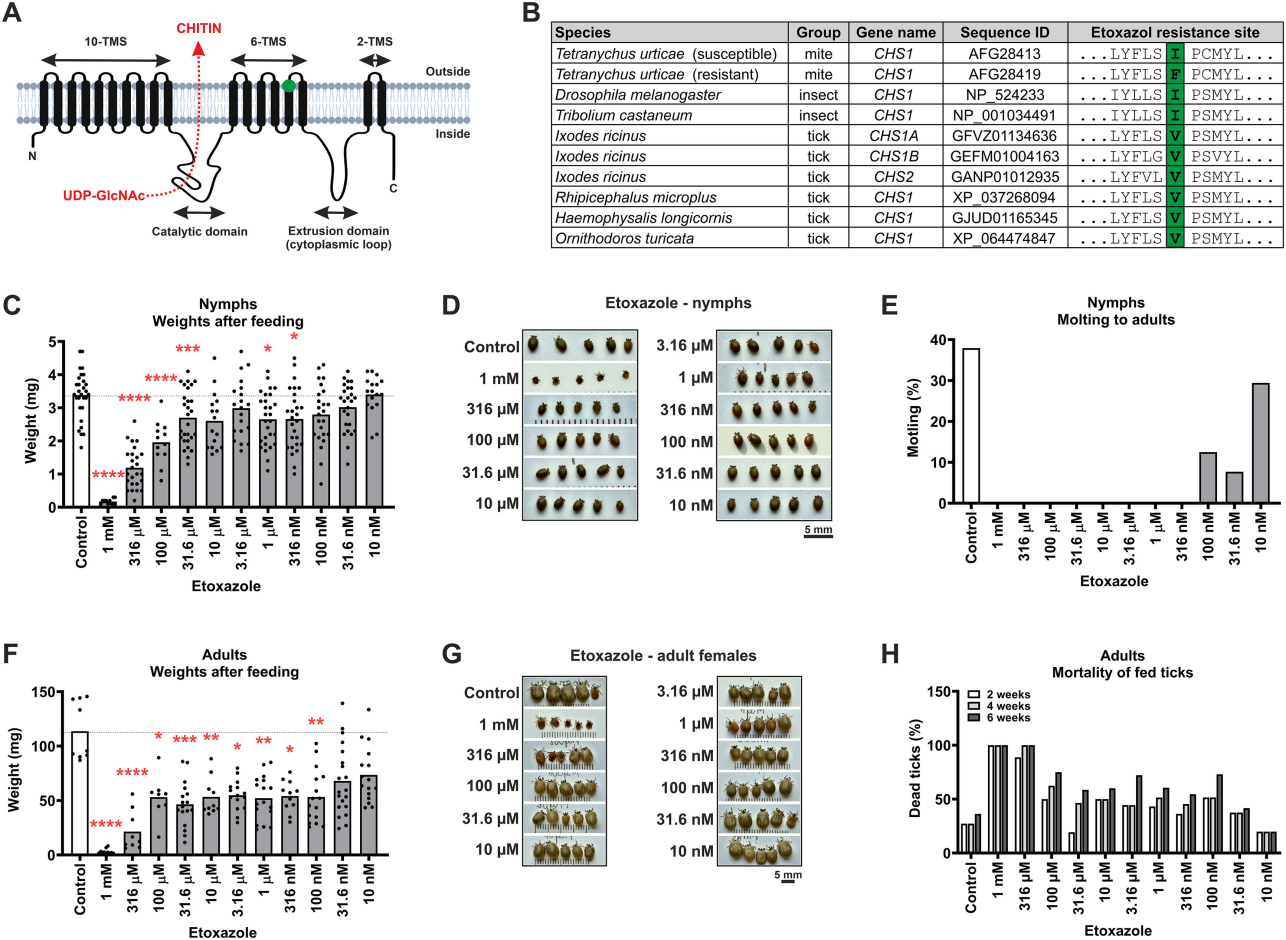
Supplementation of blood with etoxazole reduces feeding and development of ticks. **(A)** Topology model of *I. ricinus* CHS1A (GFVZ01134636) based on consensus predictions generated by TOPCONS. The green dot indicates the position (V1047) corresponding to the etoxazole resistance site I1017F previously identified in *T. urticae* CHS1 within helix 5 of the 6-TMS region (Van Leeuwen et al., 2012). **(B)** CHS amino acid sequences of mites, insects, and ticks surrounding the etoxazole resistance site (highlighted in green) corresponding to I1017F of *T. urticae* CHS1. **(C)** Weight of fully-fed nymphs fed in an artificial feeding system on bovine blood containing different concentrations of etoxazole. Ethanol (1% v/v) was used as a negative control. **(D)** Representative images of fully-fed nymphs. **(E)** Success of molting of fed nymphs to adults. **(F)** Weight of fully-fed adult females fed in an artificial feeding system on bovine blood containing different concentrations of etoxazole. **(G)** Representative images of fully-fed adult females. **(H)** Cumulative mortality of adult females at 2, 4, and 6 weeks after feeding. Results are from a single experiment, with each condition tested in two independent feeding units, each containing 12 adult females (together with 10 males to support feeding) and 25 nymphs. *P ≤ 0.05; **P ≤ 0.01; ***P ≤ 0.001; ****P ≤ 0.0001.

Etoxazole, an oxazoline-based compound, was developed in the 1980s by the Yashima company in Japan for the control of spider mites (e.g., *Tetranychus* spp.) (Tadatsu et al., 2022). Resistance to etoxazole has been linked to a point mutation in *T. urticae CHS1* (I1017F) (Douris et al., 2016) (**Figures 6A, B**). By analyzing CHS amino acid sequences from *Ixodes* and other ticks, we detected a valine at this position, a conservative, hydrophobic, nonpolar residue functionally similar to isoleucine or phenylalanine, which are found at this position in mites and insects. To assess whether etoxazole is effective against ticks, we either (i) topically applied etoxazole dissolved in ethanol to the dorsal cuticle of nymphs, followed by feeding on mice, or (ii) supplemented bovine blood with defined concentrations of etoxazole and fed nymphs and adult female *I. ricinus* through silicone membranes using an artificial feeding system.

By testing 1 mM and 31.6 µM concentrations, we found that topical application of 1 mM etoxazole to the cuticle of nymphs for 15 minutes (with the vast majority of ticks surviving these treatments) resulted in a modest but statistically significant prolongation of feeding duration and a reduction in the weight of fully-fed individuals compared to the control group (**Supplementary Figures 7A, B**). The size and general appearance of fully-fed nymphs (note that female and male nymphs are morphologically indistinguishable but differ in size (Dusbábek, 1996)) were comparable across all groups (**Supplementary Figure 7C**). However, molting success from nymph to adult was reduced by 49% in the 1 mM etoxazole group compared to the control (**Supplementary Figure 7D**).

Prior to conducting the feeding experiment with etoxazole-supplemented blood, we first examined whether etoxazole would remain stable in the blood during each 12-hour feeding period (corresponding to the regular blood exchange schedule) by adding etoxazole to fresh blood *in vitro* and incubating it at 37 °C for up to 48 hours. We measured the stability and concentration of etoxazole after 2, 4, 8, 24, and 48 hours by mass spectrometry. We found that at least 80% of the initial etoxazole concentration remained in the blood throughout the entire 48-hour period (**Supplementary Figure 1**). In subsequent feeding experiments, we then observed that etoxazole at a concentration of 1 mM (373 mg/l), introduced into the blood only after ticks had attached to the membrane, caused near-complete inhibition of successful feeding, resulting in either dead or underweight nymphs and adult females (**Figures 6C–H**; **Supplementary Figure 8**). Statistically significant reductions in weight after feeding were still observed at 316 nM in nymphs and at 100 nM in adults (**Figures 6C, F**). Only nymphs exposed to the lowest concentrations of etoxazole (100 nM, 31.6 nM, and 10 nM) or the control successfully molted to adults (**Figure 6E**). In adult females, oviposition and larval hatching were restricted to the 31.6 nM, 10 nM (3.73 µg/l), and control groups (**Figure 6H**; **Supplementary Figure 8**).

As noted above, RNAi-mediated silencing of *CHS* genes reduced chitin deposition in the cuticle and PM. To determine whether etoxazole added to the blood meal in our artificial feeding system induces a similar phenotype, we analyzed tick cuticle structure and chitin content using the same methods applied in the RNAi experiments. We selected a blood concentration of 31.6 µM etoxazole, which allowed us to obtain fully-fed nymphs of comparable weight and size (**Figure 6D**; **Supplementary Figure 9B**). Similar to the gene KDs, the endocuticle of etoxazole-treated nymphs was malformed compared to controls, significantly thinner, and filled with amorphous proteinaceous material (**Supplementary Figures 3B, 9A**). The microvilli of the epithelial cells were markedly disrupted. We also quantified chitin by immunogold electron microscopy using WGA (**Supplementary Figure 9C**) and observed a significant reduction in its abundance in the endocuticle of etoxazole-treated nymphs (**Supplementary Figures 4B**, **9D**). Quantification of GlcN released from cuticular chitin by acid hydrolysis also showed that GlcN content was significantly lower in the etoxazole-treated group (**Supplementary Figure 9E**). In summary, we showed that etoxazole treatment, like RNAi, blocks chitin synthesis and disrupts feeding and development of ticks even at nanomolar concentrations.

### 3.7 Silencing of *CHS* genes by RNAi does not block the transmission of *Borrelia*, but enhances their acquisition by ticks

*Borrelia* spirochetes are auxotrophs for GlcNAc (Fraser et al., 1997), an essential precursor for peptidoglycan synthesis, which in turn provides structural integrity, enables flagellar motility, and influences host-pathogen interactions (DeHart et al., 2021; McClune et al., 2025). *In vitro*, *Borrelia* can utilize GlcNAc monomers from the culture medium or import chitobiose (a GlcNAc dimer) via a specific chitobiose transporter (*bbb04*, *chbC*) (Tilly et al., 2001, Tilly et al., 2004). In addition, they can also utilize chitotriose, chitohexose or even crude chitin (Rhodes et al., 2010). To test whether the presence of chitin in the midgut PM (the primary site of *Borrelia* residency in ticks), which is produced *de novo* during tick feeding, affects (i) the metabolic reactivation of *Borrelia* in the midgut of infected ticks and their transmission to naïve mice, or (ii) the acquisition (uptake, survival, and replication) of *Borrelia* by uninfected nymphs feeding on infected mice, we examined these processes using our laboratory *Borrelia*-tick-mouse infection model (Jalovecka et al., 2024; Pospisilova et al., 2019).

**Figure 7:**
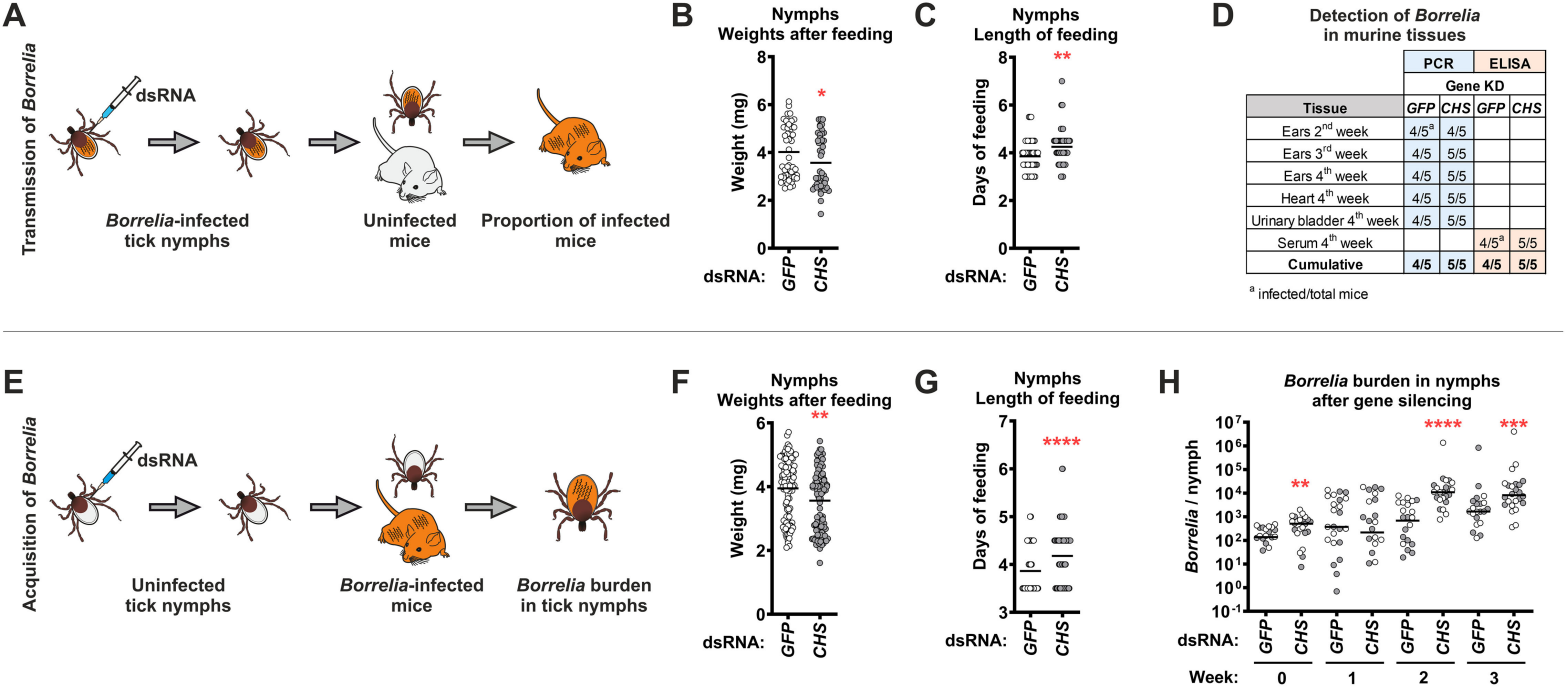
Silencing of *CHS* genes by RNAi does not block the transmission of *Borrelia* but promotes their acquisition and proliferation in ticks. **(A)** Scheme of *B. afzelii* transmission experiment. *Borrelia*-infected ticks and mice are shown in orange. **(B)** Weight of fully-fed *B. afzelii*-infected nymphs pre-injected with a mixture of *CHS* dsRNAs and fed on uninfected mice. Results are from one experiment, with each condition tested on five independent hosts (5 mice per group, 10 nymphs per mouse). **(C)** Duration of nymph feeding. **(D)** PCR detection of *B. afzelii* in mouse tissues at two, three, and four weeks after infestation, and ELISA with mouse sera collected four weeks after infestation (ELISA plates were coated with *B. afzelii* lysate). **(E)** Scheme of *B. afzelii* acquisition experiment. **(F)** Weight of fully-fed uninfected nymphs pre-injected with a mixture of *CHS* dsRNAs and fed on *B. afzelii*-infected mice. Results are from one experiment; infected nymphs were generated by feeding on six mice (6 mice per group, 20 nymphs per mouse), after which nymphs were pooled for subsequent analysis. **(G)** Duration of nymph feeding. **(H)** Absolute number (qRT-PCR) of *B. afzelii* in nymphs analyzed at 0, 1, 2, and 3 weeks after detachment (24 nymphs per time point, with equal numbers of females and males separated by weight after feeding). Female ticks are shown in gray and male ticks in white. *P ≤ 0.05; **P ≤ 0.01; ***P ≤ 0.001; ****P ≤ 0.0001.

First, we injected *B. afzelii*-infected *I. ricinus* nymphs with a mixture of *CHS1A*, *CHS1B*, and *CHS2* dsRNAs and then fed them on naïve mice. The efficacy of the triple-gene silencing was confirmed by qRT-PCR (**Supplementary Figure 10**). The fully-fed nymphs in the *CHS*s-KD group exhibited significantly lower body weights and prolonged feeding duration compared to the dsGFP controls (**Figures 7B, C**). We then followed the progression of infection in the mice for four weeks to investigate the effects of impaired tick chitin synthesis on *Borrelia* transmission. Nevertheless, all mice exposed to *CHS*-KD ticks became infected, as confirmed by PCR or ELISA of host tissues and sera (**Figure 7D**).

Second, we fed uninfected nymphs subjected to the triple *CHS* KD on *B. afzelii*-infected mice and assessed acquisition of spirochetes by quantifying *Borrelia* burden in whole nymph bodies immediately after feeding and over the following three weeks using qRT-PCR. As in the transmission experiment, *CHS*-silenced nymphs showed significantly lower body weights and longer feeding duration compared to dsGFP controls (**Figures 7F, G**). Contrary to our expectations, the *Borrelia* load in *CHS*-KD nymphs was significantly higher than in controls at 0, 2, and 3 weeks after feeding (**Figure 7H**). Overall, our results suggest that chitin, which is synthesized *de novo* by tick CHSs, is not essential for the transmission of *Borrelia* spirochetes by *I. ricinus* nymphs. In contrast, the abundance of chitin in the PM appears to limit the acquisition and subsequent multiplication of *Borrelia* within ticks.

## 4 Discussion

Chitin synthesis is a fundamental biological process in arthropods (Muthukrishnan et al., 2012, Muthukrishnan et al., 2022). While CHSs have been extensively characterized in insects, this study, conducted with the European tick *I. ricinus*, provides the first functional analysis of CHSs in ticks. Our results showed that these enzymes are important during blood feeding and development of ticks. In contrast to other arthropods, which typically have only two CHS isoforms (cuticular CHS1/CHSA and midgut CHS2/CHSB) (Rana et al., 2020), we identified three *CHS* genes in *Ixodes* ticks, *CHS1A*, *CHS1B*, and *CHS2*. Functional characterization showed that CHS1A plays a dominant role in cuticle formation, while all three CHSs contribute, directly or indirectly, to chitin deposition in the PM that forms in the midgut shortly after the beginning of blood feeding. Expression analysis confirmed this distinction, with *CHS1A* strongly expressed in the epidermis of juvenile stages (nymphs), while *CHS1B* and *CHS2* were expressed predominantly in the midgut. The marked upregulation of these genes during feeding reflects their importance at this stage and later in tick development. The predominant expression in fully-fed juvenile stages aligns with their need to molt, while the reduced expression in fully-fed adult females, who have already reconstituted their cuticle during feeding, corresponds to their transition from molting to initiating egg production. Additionally, the presence of *CHS1* transcripts in the Malpighian tubules of nymphs and adult females during and after feeding, along with *CHS1B* and *CHS2* transcripts in the ovaries (as well as in the midgut) of fully-fed adult females, suggests that chitin synthesis may play previously unexplored roles in osmoregulation, excretion, and reproduction. Further studies mapping the chitin structures in these organs could provide insights into these functions.

Silencing of *CHS*s by RNAi resulted in significant physiological defects that impaired weight, survival, molting, and oviposition and hatching efficiency in both nymphs and adults, underscoring the critical role of chitin synthesis in tick physiology during blood meal processing. Of the three *CHS* genes examined, *CHS1A*-KD impaired cuticle integrity, making ticks more fragile and significantly decreasing their ability to feed and survive after feeding. We observed that although the endocuticle, the layer of the cuticle closest to the epithelial cells, was synthesized in *CHS1A-*KD tick nymphs, it was significantly thinner and exhibited marked structural alterations compared to that of dsGFP control ticks. In untreated, unfed ticks, only a few endocuticle layers (lamellae) can be observed (Kozisek et al., 2024; Starck et al., 2018). Their number then expands extensively during blood feeding. However, in *CHS1A*-KD ticks, these lamellae were completely absent and were instead replaced by rounded, amorphous structures, likely composed of proteinaceous material secreted by the epithelial cells. This phenotype was therefore attributed to the absence of chitin in the endocuticle, which was confirmed by WGA lectin staining and mass spectrometry. The microvilli of epithelial cells in the KD group were either absent or visibly malformed (**Figure 3A**; **Supplementary Figure 2**), further indicating a profound disruption of cuticle synthesis. In contrast to the primary role of CHS1A in the cuticle, silencing of any *CHS* gene significantly reduced WGA lectin-stained chitin content in the midgut PM, a structure composed of glycoproteins associated with the chitin network. The PM of *I. ricinus* nymphs, which is not present in unfed ticks, becomes detectable approximately 18 hours after the tick attaches to the host and remains intact for at least 30 days after feeding (Zhu et al., 1991). Electron microscopy revealed no obvious differences in the presence or thickness of the PM, which likely still contained proteinaceous material secreted by midgut cells. However, KD of *CHS* genes, including those dominantly expressed in the midgut (*CHS1B* and *CHS2*), led to reduced blood uptake (*CHS1A* and *CHS2*), high post-feeding mortality, and impaired reproduction in the surviving adult female ticks (all *CHS*s), underscoring the functional importance of a well-structured PM in tick physiology. The presence and function of the PM in different tick species and families (e.g., *Ixodidae* (hard ticks; long-feeding) versus *Argasidae* (soft ticks; rapid-feeding)), including the roles of CHSs in its formation, remain poorly understood and warrant further investigation.

Given their critical physiological role, which is limited to arthropods only, CHSs represent a promising target for additional tick control strategies that could circumvent the increasing resistance to conventional, mostly neurotoxic, acaricides (Obaid et al., 2022). Our chemical inhibition experiments with etoxazole, a chitin synthesis inhibitor commonly used against plant-feeding red spider mites of the genus *Tetranychus*, confirmed the essential role of CHSs in ticks. A previous study in ticks showed that soaking *Hyalomma asiaticum* (a metastriate tick species phylogenetically distant from *Ixodes*) in an etoxazole solution negatively affected tick survival (Ren et al., 2023). However, the authors focused exclusively on the pre-feeding stage, when *CHS* genes are expressed at minimal levels. In contrast, we administered etoxazole continuously during blood feeding using a membrane feeding system (Hajdusek et al., 2023), allowing precise delivery of the compound during periods of active chitin synthesis. Under these conditions, where the ingested drug acts systemically within the tick, even nanomolar concentrations of etoxazole markedly impaired blood feeding and subsequent development, resulting in a substantially stronger phenotype than observed with topical administration. The predicted disruption of chitin synthesis by etoxazole was confirmed by microscopic visualization of endocuticle malformation, which mirrored the effects observed after KD of *CHS* genes, as well as by the absence of chitin detected through WGA lectin staining and mass spectrometry.

Although etoxazole is known to target CHSs in mites (Douris et al., 2016), CHSs have not yet been validated as molecular targets in ticks. Because resistance-associated mutations were not assessed in tick populations in this study, the following considerations are predictive and based on analogy with other arthropods. In mites and certain insects, resistance to etoxazole and to the benzoylphenylurea IGR diflubenzuron has been linked to a point mutation in *CHS1*, specifically a substitution of isoleucine with phenylalanine at the same conserved position across species (e.g., I1017F in the spider mite *T. urticae* (Douris et al., 2016) or I1043F in the mosquito *Culex pipiens* (Fotakis et al., 2020)). In ticks, we identified a valine at the homologous position, a hydrophobic aliphatic residue functionally similar to isoleucine. While speculative, this conservation suggests that analogous resistance mechanisms could, in principle, emerge in ticks under selection. Notably, although fluazuron, a benzoylphenylurea acaricide structurally related to diflubenzuron, was long considered highly effective against ticks, resistance in *R.* (*Boophilus*) *microplus* has already been reported (Ferreira et al., 2025; Reck et al., 2014). This underscores the importance of monitoring *CHS* genetic variation in tick populations, particularly in geographic regions where benzoylphenylurea-class acaricides are actively used.

Lyme borreliosis is the most common vector-borne human disease in the temperate regions of the northern hemisphere (Bush and Vazquez-Pertejo, 2018). It is caused by spirochetes of the *Borrelia burgdorferi* sensu lato complex and is transmitted by the bite of *Ixodes* ticks (Hajdušek et al., 2013). Genomic analysis of *B. burgdorferi* has shown that the spirochete lacks the biosynthetic pathway to produce GlcNAc (Fraser et al., 1997), a sugar essential for peptidoglycan biosynthesis, so *Borrelia* must obtain GlcNAc from their environment in both the vertebrate host and the tick vector (DeHart et al., 2021). *In vitro*, *Borrelia* can grow in a GlcNAc-free medium only if it is supplemented with chitobiose (a dimer of GlcNAc) (Tilly et al., 2001, Tilly et al., 2004), longer GlcNAc oligomers, or chitin (Rhodes et al., 2010). Uptake of chitobiose and GlcNAc oligomers is mediated by *chbC*, which encodes the membrane-spanning component of PTS transporter (Tilly et al., 2001, Tilly et al., 2004) and is transcriptionally regulated by the alternative sigma factor RpoS (Rhodes et al., 2009). Nevertheless, *chbC* knockouts remain fully infectious in mice, can colonize ticks, and are efficiently transmitted from infected ticks to the host (Tilly et al., 2004).

We hypothesized that the absence of chitin or GlcNAc oligomers released during PM synthesis and remodeling would affect the biology of *Borrelia* in the tick midgut and, in particular, transmission to or acquisition from the vertebrate host. However, using our *Borrelia* transmission model, we observed that *B. afzelii*-infected ticks with a triple *CHS*s KD were still able to infect mice. This result is consistent with previous work on *B. burgdorferi* B31 *chbC* mutants (Tilly et al., 2004) and suggests that the chitin synthesized *de novo* by the tick during blood feeding is not required for spirochete transmission. It further implies that *Borrelia* must obtain essential GlcNAc either directly as tick-derived monomers or from alternative sources, such as tick or host glycoproteins and glycosaminoglycans. In the next experiment, we examined whether synthesis of chitin is required for the acquisition of *Borrelia* by uninfected nymphs feeding on infected mice and for the subsequent multiplication of spirochetes in the tick midgut. Contrary to our expectations, *CHS* KD significantly increased the *Borrelia* burden in fully-fed nymphs. This elevated spirochete load remained detectable two and three weeks after feeding. We hypothesize that the increased *Borrelia* burden in *CHS*-silenced nymphs may result from several non-mutually exclusive mechanisms: (i) enhanced attraction of spirochetes to the tick feeding site (Murfin et al., 2019), (ii) reduced PM chitin content leading to altered barrier properties and/or increased access of spirochetes to the midgut epithelium, potentially facilitating tighter adhesion to epithelial receptors such as TROSPA (Pal et al., 2004), and (iii) changes in nutrient availability within the midgut lumen, such as increased access to GlcNAc monomers due to reduced incorporation into chitin. Future work could test these possibilities by localizing spirochetes relative to the PM and microvilli, quantifying PM permeability and/or spirochete adherence to the midgut epithelium under *CHS* KD conditions and directly measuring GlcNAc and related metabolites in the midgut during feeding. Collectively, our findings support a model in which PM chitin helps limit midgut colonization by *Borrelia*, highlighting how tick structural biology may shape pathogen dynamics within the vector.

In summary, we demonstrated that inhibiting CHSs in ticks, either through RNAi or chemical inhibition, disrupts not only reproduction, as previously shown in spider mites (Dekeyser, 2005; Douris et al., 2016), but also the blood-feeding process itself. These findings identify CHSs as a promising molecular target that complements current acaricides, which predominantly act on neuronal ion channels. Combining CHS inhibitors that block chitin biosynthesis and cuticle remodeling with existing compounds may reduce the required doses of active ingredients while maintaining or enhancing efficacy, thereby lowering both costs and environmental burden. Given that ticks remain attached to the host and exposed to immune components for extended periods, CHSs may also represent suitable candidates for anti-tick vaccines that impair PM integrity. Moreover, the potential for *CHS* gene mutations to confer acaricide resistance in ticks, as observed in other arthropods (Douris et al., 2016), warrants investigation and could inform molecular surveillance efforts. Incorporating CHS-targeted interventions into existing tick control strategies offers a promising avenue for more sustainable and effective management of tick populations and the diseases they transmit.

## Data Availability

The datasets presented in this study can be found in online repositories. The names of the repository/repositories and accession number(s) can be found in the article/**Supplementary Material**.
