# Supplementary material for: Disruption of chitin synthases impairs tick feeding and reproduction, validating a broad-spectrum acaricide target
Source: Front Cell Infect Microbiol. 2026 May 15;16:1822003. doi: 10.3389/fcimb.2026.1822003 (PMC13219254; doi:10.3389/fcimb.2026.1822003)
Supplement: Supplementary file 1 [file DataSheet1.pdf]

Supplementary Material

1.1 Supplementary Figures

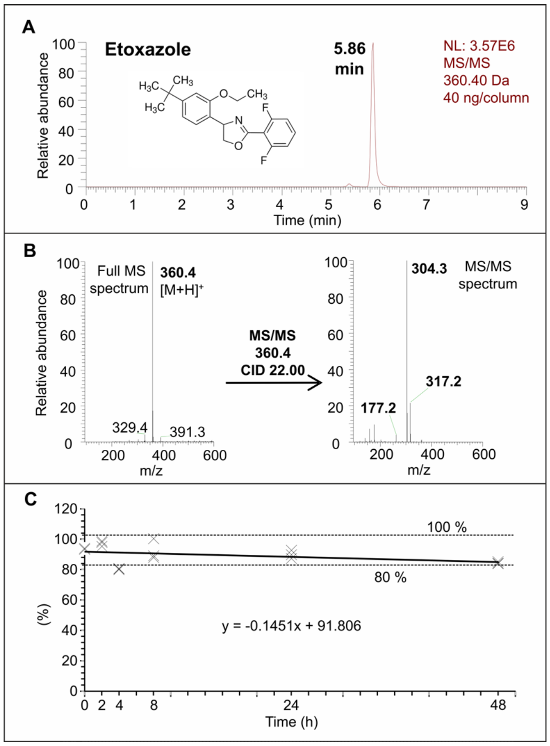

Supplementary Figure 1. Etoxazole remains stable in blood at 37 °C for at least 48 hours. **(A)** Chromatogram of etoxazole (retention time: 5.86 min). **(B)** Full MS spectrum of etoxazole, the parent ion [M+H]+ (360.4 Da) is highlighted in bold; the MS/MS spectrum of 360.4 Da [M+H]+, with highlighted diagnostic/daughter ions (317.2 Da, 304.3, and 177.2 Da). **(C)** 48-hour stability curve of etoxazole in blood (technical triplicates).

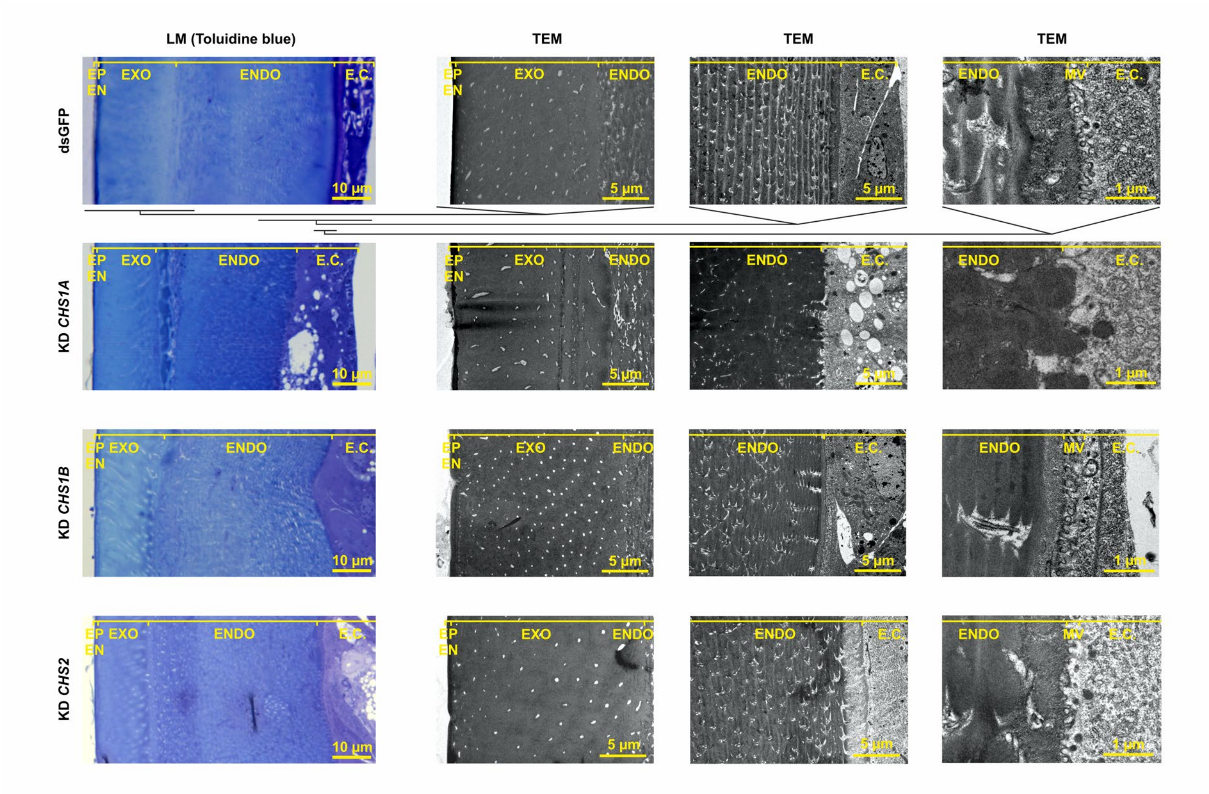

Supplementary Figure 2. Silencing of the *CHS1A* gene by RNAi alters the morphology of the tick cuticle. Representative microscopy images showing the structure of the tick cuticle in the dsGFP control, *CHS1A*-, *CHS1B*-, and *CHS2*-KD nymphs one week after feeding. Light microscopy (LM), transmission electron microscopy (TEM), epithelial cells (EC), endocuticle (ENDO), exocuticle (EXO), epicuticle (EP), envelope (EN), microvilli (MV). The horizontal bars beneath the dsGFP LM image indicate the cuticle regions selected for subsequent TEM imaging; comparable anatomical regions were consistently selected for TEM across all groups.

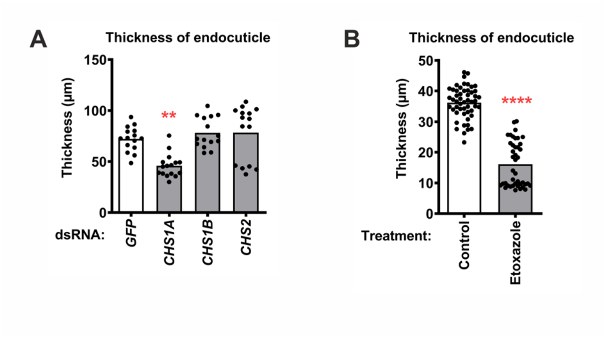

Supplementary Figure 3. Silencing of the *CHS1A* gene by RNAi or supplementation of blood with etoxazole reduces thickness of the tick endocuticle in fully-fed nymphs. Thickness of the endocuticle in **(A)** the dsGFP control and *CHS1A*-KD groups, and **(B)** the ethanol control (1% v/v) and etoxazole-treated group (31.6 µM) was measured in ImageJ. Each dot represents the measured thickness of **(A)** five sampled areas per section across three individual ticks per group and **(B)** at least 10 sampled areas per section across three individual ticks per group collected from the same feeding experiment. $**P \leq 0.01$; $****P \leq 0.0001$.

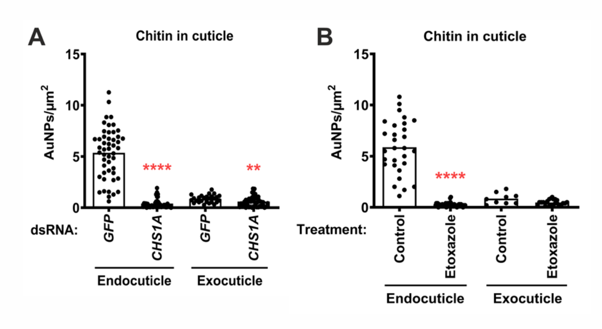

Supplementary Figure 4. Silencing of the *CHS1A* gene by RNAi or supplementation of blood with etoxazole both reduce chitin content in the tick cuticle of fully-fed nymphs. Labeling density (LD) of WGA staining (gold particles/µm² cuticle) for **(A)** the dsGFP control and *CHS1A*-KD groups, and **(B)** the ethanol control (1% v/v) and etoxazole-treated group (31.6 µM). Each dot represents the LD value of at least 10 sampled areas per section, across three individual ticks per group collected from the same feeding experiment. Gold nanoparticles (AuNPs). $^{**}P \leq 0.01$; $^{****}P \leq 0.0001$.

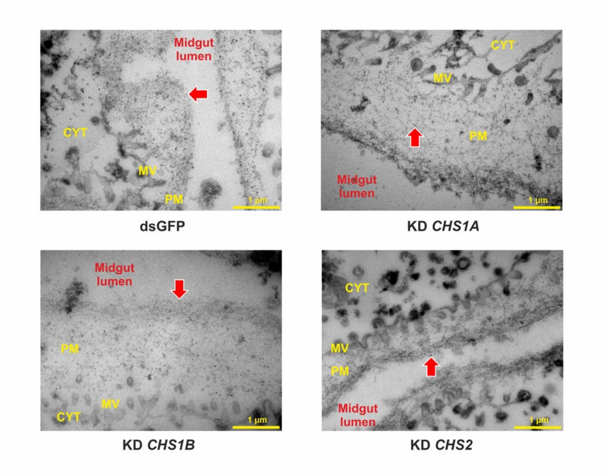

Supplementary Figure 5. Silencing of the individual *CHS* genes by RNAi reduces the chitin content in the PM of the midgut. Representative TEM images showing WGA-gold labeling in the PM of fully-fed nymph midguts for each KD group; red arrows indicate the gold particles. Cytoplasm (CYT), microvilli (MV), peritrophic matrix (PM), wheat germ agglutinin (WGA).

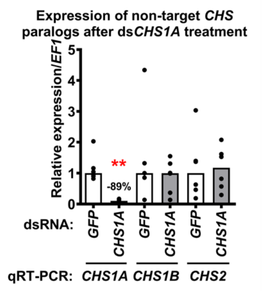

Supplementary Figure 6. Silencing of *CHS1A* by RNAi does not alter the expression of midgut-specific *CHS* paralogs. Efficacy of gene silencing in fully-fed nymphs (whole bodies) measured by qRT-PCR. Each dot represents one pool of five nymphs (five biological replicates), and medians are plotted.

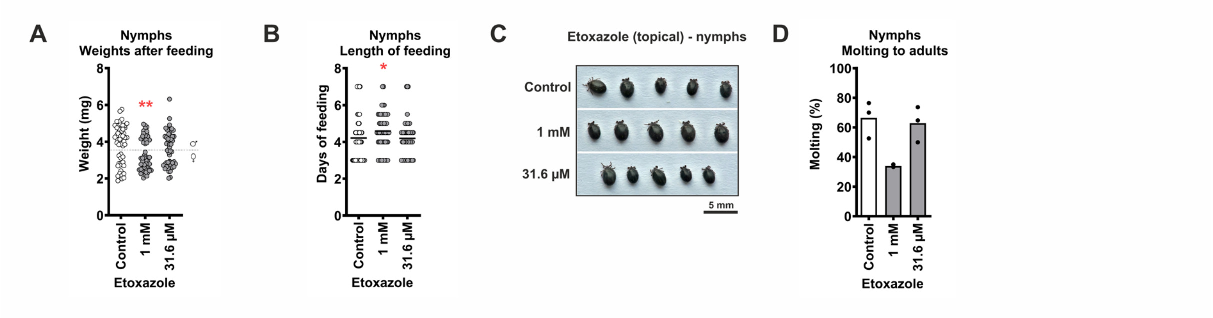

Supplementary Figure 7. Topical application of etoxazole reduces feeding and development of tick nymphs. Etoxazole, dissolved in 100% ethanol, was topically applied to the dorsal cuticle of nymphs, followed by feeding on mice. Ticks treated with 100% ethanol served as a negative control. **(A)** Weight of fully-fed nymphs pre-treated with etoxazole. Results are from a single experiment, with each condition tested on three independent hosts (3 mice per group, 20 nymphs per mouse). **(B)** Duration of nymph feeding. **(C)** Representative images of fully-fed nymphs. **(D)** Success of molting of fed nymphs to adults. *P ≤ 0.05; **P ≤ 0.01.

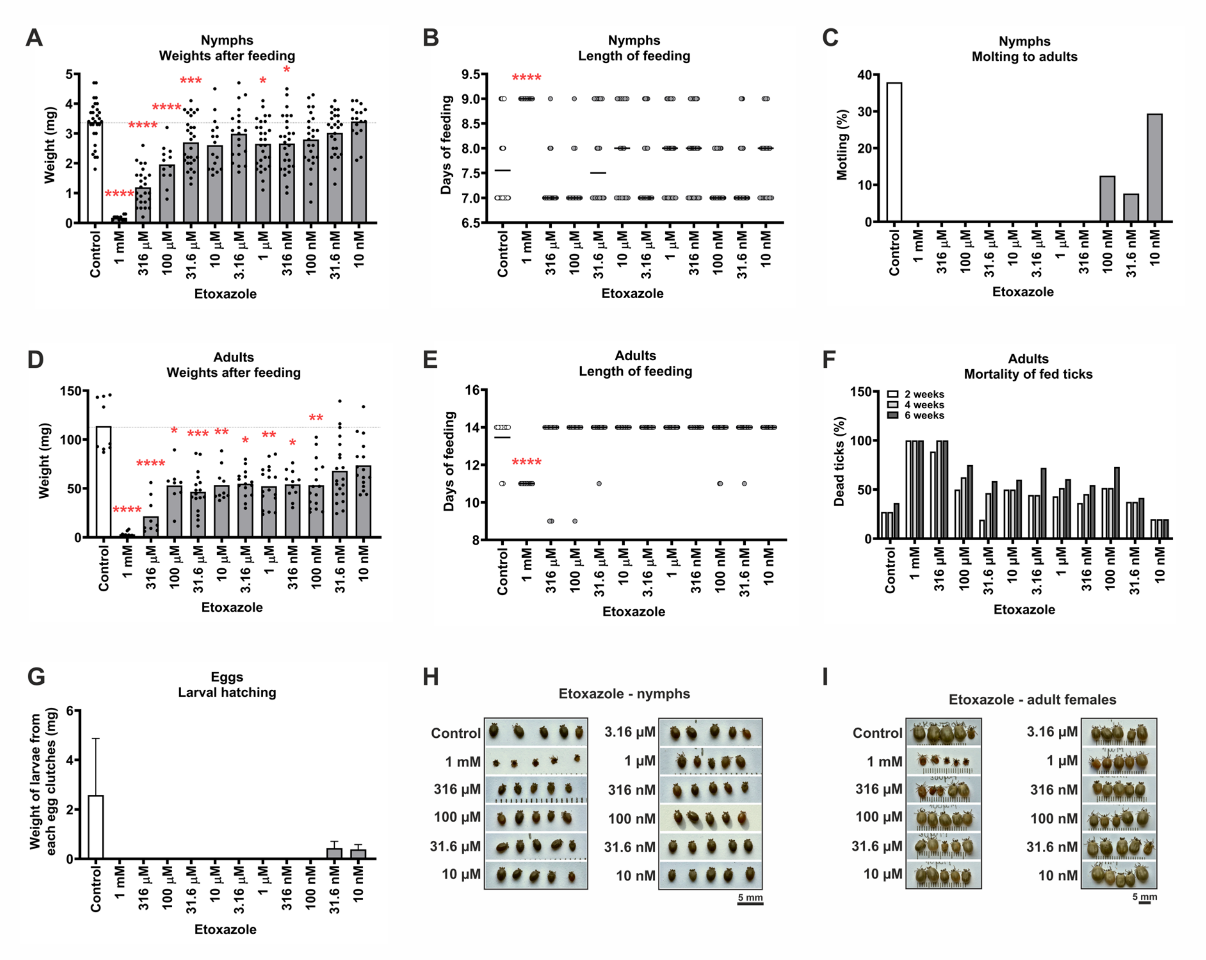

Supplementary Figure 8. Etoxazole in blood reduces feeding and development of ticks. **(A)** Weight of fully-fed nymphs fed in an artificial feeding system on bovine blood containing different concentrations of etoxazole. Ethanol was used as a negative control. **(B)** Duration of nymph feeding. **(C)** Success of molting of fed nymphs to adults. **(D)** Weight of fully-fed adult females fed in an artificial feeding system on bovine blood containing different concentrations of etoxazole. **(E)** Duration of feeding of adult females. **(F)** Cumulative mortality of adult females at 2, 4, and 6 weeks after feeding. **(G)** Total weight of all larvae hatched from the individual egg clutches. Each dot represents one clutch. The results were expressed as the mean ± standard error (SEM). **(H)** Representative images of fully-fed nymphs. **(I)** Representative images of fully-fed adult females. Results are from a single experiment, with each condition tested in two independent feeding units, each containing 12 adult females (together with 10 males to support feeding) and 25 nymphs. $*P \le 0.05$; $**P \le 0.01$; $***P \le 0.001$; $****P \le 0.0001$.

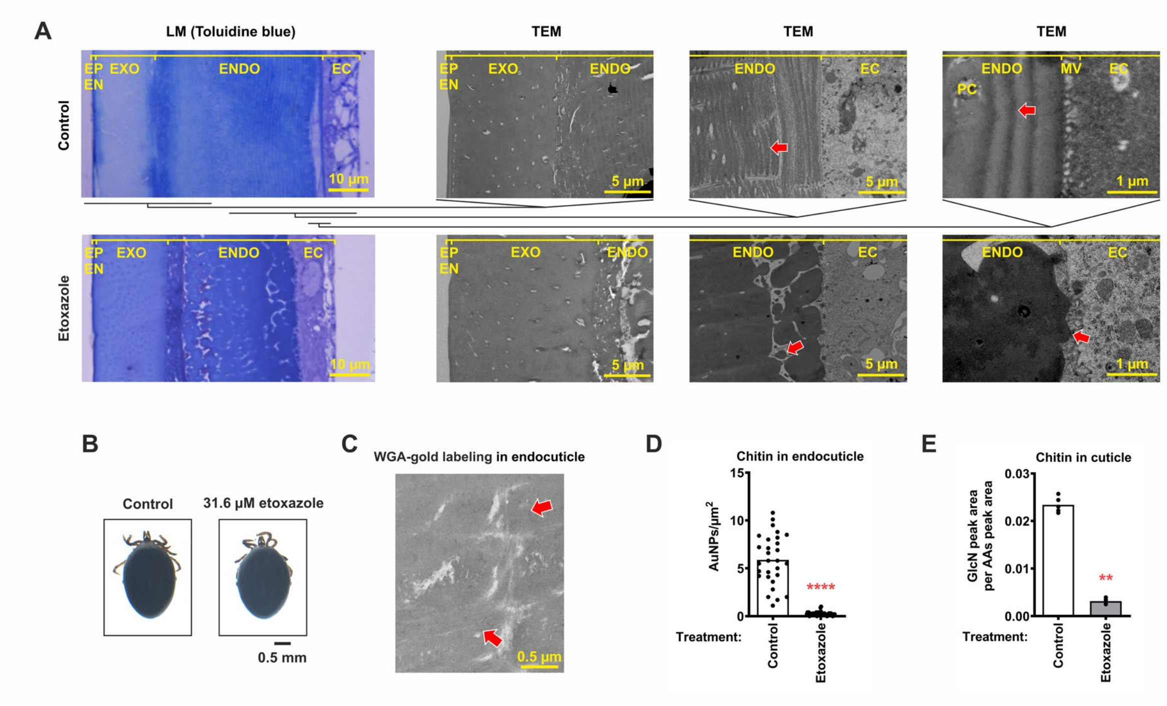

Supplementary Figure 9. Etoxazole in blood alters the morphology of the tick cuticle. **(A)** Representative microscopy images showing the structure of the cuticles from fully-fed nymphs (one week after feeding) that were fed in an artificial feeding system on bovine blood supplemented with 31.6 µM etoxazole. Ethanol (1% v/v) was used as a negative control. Light microscopy (LM), transmission electron microscopy (TEM), epithelial cells (EC), endocuticle (ENDO), exocuticle (EXO), epicuticle (EP), envelope (EN), microvilli (MV), pore canals (canaliculi, PC). Red arrows show the lamellar structure of the endocuticle in control nymphs compared to the disrupted endocuticle in the etoxazole-treated group. The horizontal bars beneath the control LM image indicate the cuticle regions selected for subsequent TEM imaging; comparable anatomical regions were consistently selected for TEM across all groups. **(B)** Representative images of control and etoxazole-treated nymphs used for the analyses. Nymphs of comparable weight and size were selected from both groups. **(C)** Representative TEM image of WGA-gold labeling (red arrows) in the endocuticle of control nymphs, illustrating the labeling signal quantified in panel **(D)**. **(D)** Labeling density (LD) of WGA staining (gold particles/µm² cuticle) in control and etoxazole-treated groups. Each dot represents the LD value of at least 10 sampled areas per section, across three individual ticks per group collected from the same feeding experiment. Gold nanoparticles (AuNPs). **(E)** Relative amounts of glucosamine (GlcN; the end product of chitin hydrolysis) in the cuticles of fully-fed nymphs quantified by liquid chromatography-mass spectrometry (LC-MS) and normalized to the amino acid (AA) content of each sample. $*P \leq 0.05$; $****P \leq 0.0001$.

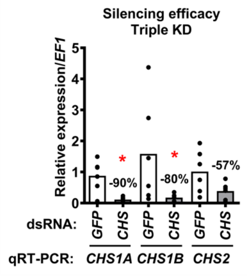

Supplementary Figure 10. Efficacy of gene silencing with a mixture of *CHS1A*, *CHS1B*, and *CHS2* dsRNAs in fully-fed nymphs (whole bodies) measured by qRT-PCR. Each sample represents a pool of five nymphs. Each dot represents one pool of five nymphs (five biological replicates), and medians are plotted.
