# Supplementary material for: Disruption of chitin synthases impairs tick feeding and reproduction, validating a broad-spectrum acaricide target
Source: Front Cell Infect Microbiol. 2026 May 15;16:1822003. doi: 10.3389/fcimb.2026.1822003 (PMC13219254; doi:10.3389/fcimb.2026.1822003)
Supplement: Supplementary file 2 [file Table1.docx]

**Supplementary Table 1.** List of primers and probes. Restriction sites for ApaI / XbaI are underlined.

| **Method** | **Target** | **NCBI reference** | **ID** | **Sequence (5´-3´)** |
| --- | --- | --- | --- | --- |
| RNAi | *Ixodes ricinus*: *chitin synthase 1A* (*CHS1A*) | GIXL01010889 | IR1291 | ATGGGCCCCCAACATCACGTTCATACC |
|  |  |  | IR1292 | ATTCTAGAGGTCTGCATCTTGCTGTGG |
|  | *I. ricinus*: *chitin synthase 1B* (*CHS1B*) | GEFM01004163 | IR1315 | ATGGGCCCGCTGCGTGCTGTGTAGTCC |
|  |  |  | IR1316 | ATTCTAGACCACCATCACTAGCAACGC |
|  | *I. ricinus*: *chitin synthase 2* (*CHS2*) | GEFM01002933 | IR1312 | ATGGGCCCGGTCGCATGTGGAACACGTG |
|  |  |  | IR1313 | ATTCTAGACTCCTTCGAGCGATTTGGTC |
| qRT-PCR (cDNA) | *Ixodes ricinus*: *chitin synthase 1A* (*CHS1A*) | GIXL01010889 | IR1289 | CGTCACCGAAACAACAACC |
|  |  |  | IR1290 | TCAGGCTCTGTCTTGCTGAG |
|  | *I. ricinus*: *chitin synthase 1B* (*CHS1B*) | GEFM01004163 | IR1712 | GAATGCATCCAATGGCCGTC |
|  |  |  | IR1713 | GCTGTGTGTTTGTCTCTGGC |
|  | *I. ricinus*: *chitin synthase 2* (*CHS2*) | GEFM01002933 | IR1307 | GGCAAGGTACCAGGAAGATG |
|  |  |  | IR1308 | GGTCGGGAACTACTGCTCAC |
|  | *I. ricinus*: *elongation factor* | GU074769 | IR524 | acgaggctctgacggaag |
|  |  |  | IR525 | cacgacgcaactccttcac |
| qRT-PCR (DNA) | *Borrelia* spp. *flagellin* | (Schwaiger et al., 2001) | IR1345 | AGCAAATTTAGGTGCTTTCCAA |
|  |  |  | IR1346 | GCAATCATTGCCATTGCAGA |
|  |  |  | Fla Probe1 | TGCTACAACCTCATCTGTCATTGTAGCATCTTTTATTTG |
|  | *Mus musculus*: *actin* | (Dai et al., 2009) | MM-ACT-F | AGAGGGAAATCGTGCGTGAC |
|  |  |  | MM-ACT-R | CAATAGTGATGACCTGGCCGT |
|  |  |  | MM-ACT-PROBE | CACTGCCGCATCCTCTTCCTCCC |
| PCR | *Borrelia* spp. *flagellin* | (Schwaiger et al., 2001) | IR1345 | AGCAAATTTAGGTGCTTTCCAA |
|  |  |  | IR1346 | GCAATCATTGCCATTGCAGA |
|  | *I. ricinus*: *actin* | AJ889837 | IR1558 | CTACGAAGGGTACGCTCTGC |
|  |  |  | IR1559 | GACTCGTCGTATTCCTGCTTG |
|  | *M. musculus*: *actin* | (Dai et al., 2009) | MM-ACT-F | AGAGGGAAATCGTGCGTGAC |
|  |  |  | MM-ACT-R | CAATAGTGATGACCTGGCCGT |

Dai, J., Wang, P., Adusumilli, S., Booth, C. J., Narasimhan, S., Anguita, J., et al. (2009). Antibodies against a tick protein, Salp15, protect mice from the Lyme disease agent. *Cell Host Microbe* 6, 482–492. doi:10.1016/j.chom.2009.10.006.

Schwaiger, M., Peter, O., and Cassinotti, P. (2001). Routine diagnosis of *Borrelia burgdorferi* (sensu lato) infections using a real-time PCR assay. *Clin. Microbiol. Infect.* 7, 461–469. doi:10.1046/j.1198-743x.2001.00282.x.
